# Plasma aldosterone concentrations elevation in hypertensive patients: the dual impact on hyperuricemia and gout

**DOI:** 10.3389/fendo.2024.1424207

**Published:** 2024-07-30

**Authors:** Shuaiwei Song, Xintian Cai, Junli Hu, Qing Zhu, Di Shen, Huimin Ma, Yingying Zhang, Rui Ma, Pan Zhou, Wenbo Yang, Jing Hong, Delian Zhang, Nanfang Li

**Affiliations:** ^1^ Key Laboratory of Xinjiang Uygur Autonomous Region “Hypertension Research Laboratory”, Hypertension Center of People’s Hospital of Xinjiang Uygur Autonomous Region, Xinjiang Hypertension Institute, Xinjiang Clinical Medical Research Center for Hypertension (Cardio-Cerebrovascular) Diseases, Urumqi, China; ^2^ NHC Key Laboratory of Hypertension Clinical Research, Hypertension Center of People’s Hospital of Xinjiang Uygur Autonomous Region, Xinjiang Hypertension Institute, Xinjiang Clinical Medical Research Center for Hypertension (Cardio-Cerebrovascular) Diseases, Urumqi, China

**Keywords:** plasma aldosterone concentration, hypertension, uric acid, hyperuricemia, gout

## Abstract

**Background:**

Prior research has highlighted the association between uric acid (UA) and the activation of the renin-angiotensin-aldosterone system (RAAS). However, the specific relationship between aldosterone, the RAAS’s end product, and UA-related diseases remains poorly understood. This study aims to clarify the impact of aldosterone on the development and progression of hyperuricemia and gout in hypertensive patients.

**Methods:**

Our study involved 34534 hypertensive participants, assessing plasma aldosterone concentration (PAC)’s role in UA-related diseases, mainly hyperuricemia and gout. We applied multiple logistic regression to investigate the impact of PAC and used restricted cubic splines (RCS) for examining the dose-response relationship between PAC and these diseases. To gain deeper insights, we conducted threshold analyses, further clarifying the nature of this relationship. Finally, we undertook subgroup analyses to evaluate PAC’s effects across diverse conditions and among different subgroups.

**Results:**

Multivariate logistic regression analysis revealed a significant correlation between the occurrence of hyperuricemia and gout and the elevation of PAC levels. Compared to the first quartile (Q1) group, groups Q2, Q3, and Q4 all exhibited a significantly increased risk of occurrence. Moreover, the conducted RCS analysis demonstrated a significant nonlinear dose-response relationship, especially when PAC was greater than 14 ng/dL, with a further increased risk of hyperuricemia and gout. Finally, comprehensive subgroup analyses consistently reinforced these findings.

**Conclusion:**

This study demonstrates a close association between elevated PAC levels and the development of UA-related diseases, namely hyperuricemia and gout, in hypertensive patients. Further prospective studies are warranted to confirm and validate this relationship.

## Highlights

- This study is the first to determine the relationship between plasma aldosterone concentration and uric acid and uric acid-related diseases in hypertensive patients.
- Plasma aldosterone concentrations in hypertensive patients were independently associated with the risk of uric acid-related diseases such as hyperuricemia and gout.
- Dose-response relationship: Plasma aldosterone concentrations greater than 14 ng/dL were associated with a significantly increased risk of hyperuricemia and gout.

## 1 Introduction

Uric acid (UA) is a metabolite of purines in humans and is normally excreted through the kidneys (1). When UA is produced in excess or excreted insufficiently, it leads to an increase in the amount of UA in the blood, forming hyperuricemia (2). Hyperuricemia, as a metabolic disorder, has attracted widespread attention among hypertensive patients (3–5). The increase in UA levels is not only closely related to the occurrence of gout but also to hypertension, atherosclerosis, diabetes, and other diseases (3, 5–7). Especially in recent years, with changes in lifestyle, the incidence of hypertension and hyperuricemia has increased year by year, becoming a major public health problem worldwide (8, 9). Consequently, understanding the interplay between hypertension and UA-related diseases such as hyperuricemia and gout is of broad interest.

Most previous studies on the causes of hyperuricemia and gout have focused on factors such as chronic high-purine diets, renal excretory dysfunction, excessive obesity, and genetic predisposition (10–13). Remarkably, the role of aldosterone, an important mineral corticosteroid regulating water and salt balance and blood pressure, has been largely overlooked (14). Research has shown that the activation of the renin-angiotensin-aldosterone system (RAAS) is linked not only to hypertension but may also influence UA metabolism (15–18). In patients with atrial fibrillation (AF), for instance, UA levels were associated with RAAS activation, indicating that renin activity and plasma aldosterone concentration (PAC) levels are positively correlated with UA levels (16). Moreover, PAC was found to affect UA concentration and urinary potassium excretion, demonstrating a direct relationship between urinary potassium levels and UA concentration (18). However, contrasting findings from Mulè G et al. suggest that this relationship may not be straightforward, as their study observed higher UA levels with higher PAC, but the significance of these findings diminished after adjusting for certain factors (19). Therefore, given the controversial nature of the relationship between PAC and UA and the complexity and harmful effects of hypertension, aldosterone, and UA-related diseases, further research is warranted.

Therefore, to address the ongoing debate on the relationship between PAC and UA levels in hypertensive patients, as well as to explore previously unstudied associations with hyperuricemia and gout, we designed a comprehensive cross-sectional study. Our objective is to investigate the connection between PAC levels and UA-related diseases like hyperuricemia and gout in this specific population. Through this research, we aim to identify new opportunities for the diagnosis and treatment of these conditions.

## 2 Materials and methods

### 2.1 Study population

#### 2.1.1 Inclusion criteria

The study cohort was derived from patients attending the Xinjiang Hypertension Center between January 2014 and December 2023. Over this period, a total of 41131 individuals received a diagnosis of hypertension. Of these, 40202 individuals underwent PAC testing.

#### 2.1.2 Exclusion criteria

**Figure 1:**
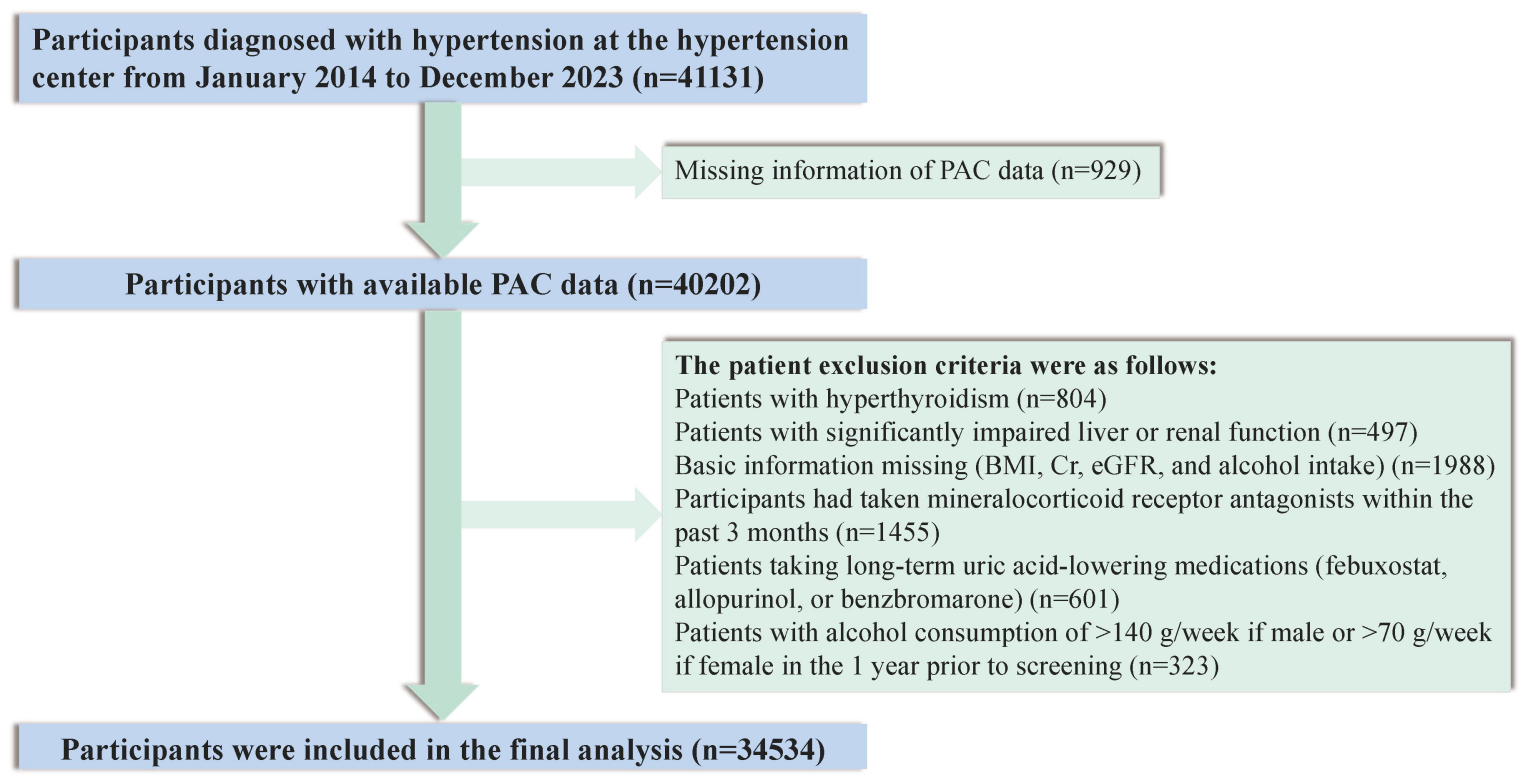
Flow chart of participant selection.

To ensure study reliability, we initially excluded participants lacking essential baseline data pertinent to UA metabolism, including body mass index (BMI), serum creatinine (Cr) levels, estimated glomerular filtration rate (eGFR), and alcohol consumption patterns. Subsequently, we excluded individuals with markedly compromised liver or renal function, hyperthyroidism, or those with a history of heavy alcohol consumption. Participants who had recently received mineralocorticoid receptor antagonists within the preceding three months or who had a documented history of chronic use of UA-lowering medications were also excluded from the study. Following the application of these stringent exclusion criteria, a final study population of 34534 participants was enrolled (**Figure 1**).

This study received approval from the Research Ethics Committee of the Xinjiang Uygur Autonomous Region People’s Hospital (Approval No. KY2022080905). All procedures adhered to the ethical standards outlined in the Declaration of Helsinki, and all participants provided their informed written consent prior to study commencement.

### 2.2 Data collection and definitions

Participants’ demographic information, clinical history, lifestyle, physical examination, medication history, and laboratory data were collected through the hospital’s electronic medical record system. **Supplementary Table S1** shows the types and names of medications used by all participants. Specific measurements and calculations of height, weight, smoking status, alcohol consumption, blood pressure, and BMI are detailed in the **Supplementary Material**. Laboratory tests included results of alanine aminotransferase (ALT), aspartate aminotransferase (AST), Cr, blood urea nitrogen (BUN), total cholesterol (TC), triglycerides (TG), high-density lipoprotein (HDL-C), low-density lipoprotein cholesterol (LDL-C), fasting blood glucose (FPG), glycosylated hemoglobin (HbA1c), and UA, all measured by an automatic biochemical analyzer. The PAC was measured by radioimmunoassay (DSL-8600; DSL, Webster, TX). The eGFR was calculated by the CKD-EPI equation. All hormone measurements are based on current guidelines and previous studies published by our team (20–23). The specific measurement methods and the definitions of the various diseases are given in detail in the **Supplementary Material**.

### 2.3 Main outcomes

The primary outcomes of our study were the incidence of UA-related conditions: hyperuricemia and gout. Hyperuricemia was diagnosed in accordance with the criteria established by the Chinese Endocrine Association and the International Endocrine Association. Specifically, a diagnosis of hyperuricemia is warranted when serum UA levels exceed 420 μmol/L (7.0 mg/dL) in males and 360 μmol/L (6.0 mg/dL) in females, as referenced in the literature (24–26). The identification of gout was guided by the International Rheumatic Association’s standards. A confirmed diagnosis of gout was contingent upon the evaluation of UA levels, articular symptoms, radiographic findings from plain X-rays, and results from joint aspiration (27–29).

### 2.4 Statistical analysis

Participants were categorized into quartiles based on PAC. To compare between groups, we employed the χ^2^ test for categorical variables. For continuous variables, we initially assessed normality. For those adhering to a normal distribution, we utilized univariate analysis of variance (ANOVA). Conversely, for those not following a normal distribution, we employed the Kruskal-Wallis test. Multicollinearity among predictors was evaluated using the variance inflation factor (VIF), with a threshold VIF value of less than 10 indicating the absence of multicollinearity, as presented in **Supplementary Table S2** and depicted in **Supplementary Figure S1**.

To elucidate the impact of PAC on the development of UA-related diseases, namely hyperuricemia, and gout, we employed multifactorial logistic regression analysis to construct five distinct models. These models were designed to ascertain the independent association of PAC with these conditions. Furthermore, to delineate the dose-response relationship, we utilized restricted cubic spline (RCS) curves and conducted threshold analysis centered on the turning points identified within the RCS analysis.

In a bid to bolster the reliability of our findings, we undertook a comprehensive subgroup and sensitivity analysis. Please refer to the **Supplementary Materials** for a detailed description of the statistical analysis.

All data analyses were conducted using R software, version 4.2.2. Statistical significance was defined as a two-sided P-value of less than 0.05.

## 3 Results

### 3.1 Characteristics of participants

This study enrolled a total of 34534 participants, and their baseline characteristics, stratified by PAC quartiles, are presented in **Table 1**. The average age of the participants was 51.10 years, with a majority male composition, representing 56.91% of the study population. Participants in the higher PAC quartiles were found to be relatively younger and exhibited lower smoking prevalence compared to those in the lowest quartile (Q1).

**Table 1:** Characteristics of the study population based on PAC quartiles.

Characteristic	Q1	Q2	Q3	Q4	P value
PAC (ng/dL)	<11.58	11.58-14.16	14.16-18.81	>18.81	
N	8636	8636	8636	8635	
Age (years)	52.29 ± 12.88	51.64 ± 11.80	50.78 ± 12.37	49.69 ± 12.43	<0.001
Sex					0.034
Female	3678 (42.59%)	3727 (43.16%)	3654 (42.31%)	3830 (44.35%)	
Male	4958 (57.41%)	4909 (56.84%)	4982 (57.69%)	4805 (55.65%)	
BMI (kg/m^2^)	26.88 ± 3.67	26.91 ± 3.63	26.93 ± 3.61	26.97 ± 3.61	0.391
SBP (mmHg)	146.19 ± 18.46	146.00 ± 18.19	145.86 ± 18.24	146.01 ± 18.19	0.695
DBP (mmHg)	88.31 ± 13.73	88.11 ± 13.57	87.86 ± 13.47	88.11 ± 13.60	0.185
Current smoking (%)	3042 (35.22%)	2936 (34.00%)	2925 (33.87%)	2590 (29.99%)	<0.001
Medical history					
PA (%)	952 (11.02%)	1280 (14.82%)	1410 (16.33%)	1567 (18.15%)	<0.001
Diabetes (%)	1488 (17.23%)	1369 (15.85%)	1292 (14.96%)	1381 (15.99%)	<0.001
CHD (%)	918 (10.63%)	780 (9.03%)	731 (8.46%)	753 (8.72%)	<0.001
Cancer (%)	68 (0.79%)	125 (1.45%)	100 (1.16%)	211 (2.44%)	<0.001
Laboratory tests					
ALT (U/L)	27.22 ± 17.57	26.90 ± 17.47	26.91 ± 17.46	27.05 ± 17.43	0.594
AST (U/L)	20.99 ± 8.14	20.88 ± 8.17	20.86 ± 7.99	20.93 ± 8.17	0.715
Cr (umol/L)	64.95 ± 14.63	65.08 ± 14.53	65.00 ± 14.58	65.51 ± 14.79	0.044
eGFR (ml/min/1.73 m^2^)	114.19 ± 27.77	115.27 ± 25.93	116.50 ± 27.15	117.66 ± 27.72	<0.001
BUN (mmol/L)	5.05 ± 1.35	5.04 ± 1.33	5.05 ± 1.36	5.07 ± 1.36	0.559
TC (mmol/L)	4.52 ± 0.96	4.49 ± 0.94	4.53 ± 0.96	4.51 ± 0.95	0.096
TG (mmol/L)	1.74 ± 0.90	1.73 ± 0.89	1.74 ± 0.89	1.75 ± 0.90	0.562
HDL.C (mg/dL)	1.06 ± 0.25	1.05 ± 0.24	1.06 ± 0.25	1.05 ± 0.25	0.115
LDL.C (mg/dL)	2.74 ± 0.83	2.74 ± 0.83	2.77 ± 0.81	2.77 ± 0.82	0.002
FPG (mmol/L)	5.07 ± 1.12	5.04 ± 1.09	5.02 ± 1.08	5.06 ± 1.12	0.018
HbA1c (%)	6.00 ± 0.87	5.95 ± 0.84	5.90 ± 0.81	5.91 ± 0.85	<0.001
UA (umol/L)	302.20 ± 110.89	301.40 ± 104.00	306.88 ± 103.79	320.57 ± 114.02	<0.001
PAC (ng/dL)	10.15 ± 1.54	12.90 ± 0.65	16.28 ± 1.34	23.86 ± 4.10	<0.001
Medications					
Statins (%)	1080 (12.51%)	998 (11.56%)	959 (11.10%)	900 (10.42%)	<0.001
Aspirins (%)	1100 (12.74%)	1033 (11.96%)	933 (10.80%)	906 (10.49%)	<0.001
Diuretics (%)	879 (10.18%)	872 (10.10%)	938 (10.86%)	1024 (11.86%)	<0.001
Beta-blockers (%)	1641 (19.00%)	1514 (17.53%)	1457 (16.87%)	1504 (17.42%)	0.002
Calcium channel blockers (%)	4425 (51.24%)	4427 (51.26%)	4536 (52.52%)	4836 (56.00%)	<0.001
ACEIs/ARBs (%)	4162 (48.19%)	3946 (45.69%)	3874 (44.86%)	3943 (45.66%)	<0.001
Oral hypoglycemic agents (%)	786 (9.10%)	700 (8.11%)	596 (6.90%)	639 (7.40%)	<0.001
Hyperuricemia (%)	1470 (17.02%)	1767 (20.46%)	2258 (26.15%)	2565 (29.70%)	<0.001
Gout (%)	81 (0.94%)	133 (1.54%)	184 (2.13%)	255 (2.95%)	<0.001

Data are presented as mean ± standard deviation, median (interquartile range), or as numbers, and percentages.

BMI, body mass index; SBP, systolic blood pressure; DBP, diastolic blood pressure; PA, primary aldosteronism; CHD, coronary heart disease; ALT, alanine transaminase; AST, aspartate transaminase; Cr, creatinine; eGFR, estimated glomerular filtration rate; BUN, blood urea nitrogen; TC, total cholesterol; TG, triglyceride; HDL-C, high-density lipoprotein cholesterol; LDL-C, low-density lipoprotein cholesterol; FPG, fasting plasma glucose; HbA1c, glycosylated hemoglobin; UA, Uric acid; PAC, plasma aldosterone concentration; ARBs, angiotensin receptor blockers; ACEIs, angiotensin-converting enzyme inhibitors.

**Figure 2:**
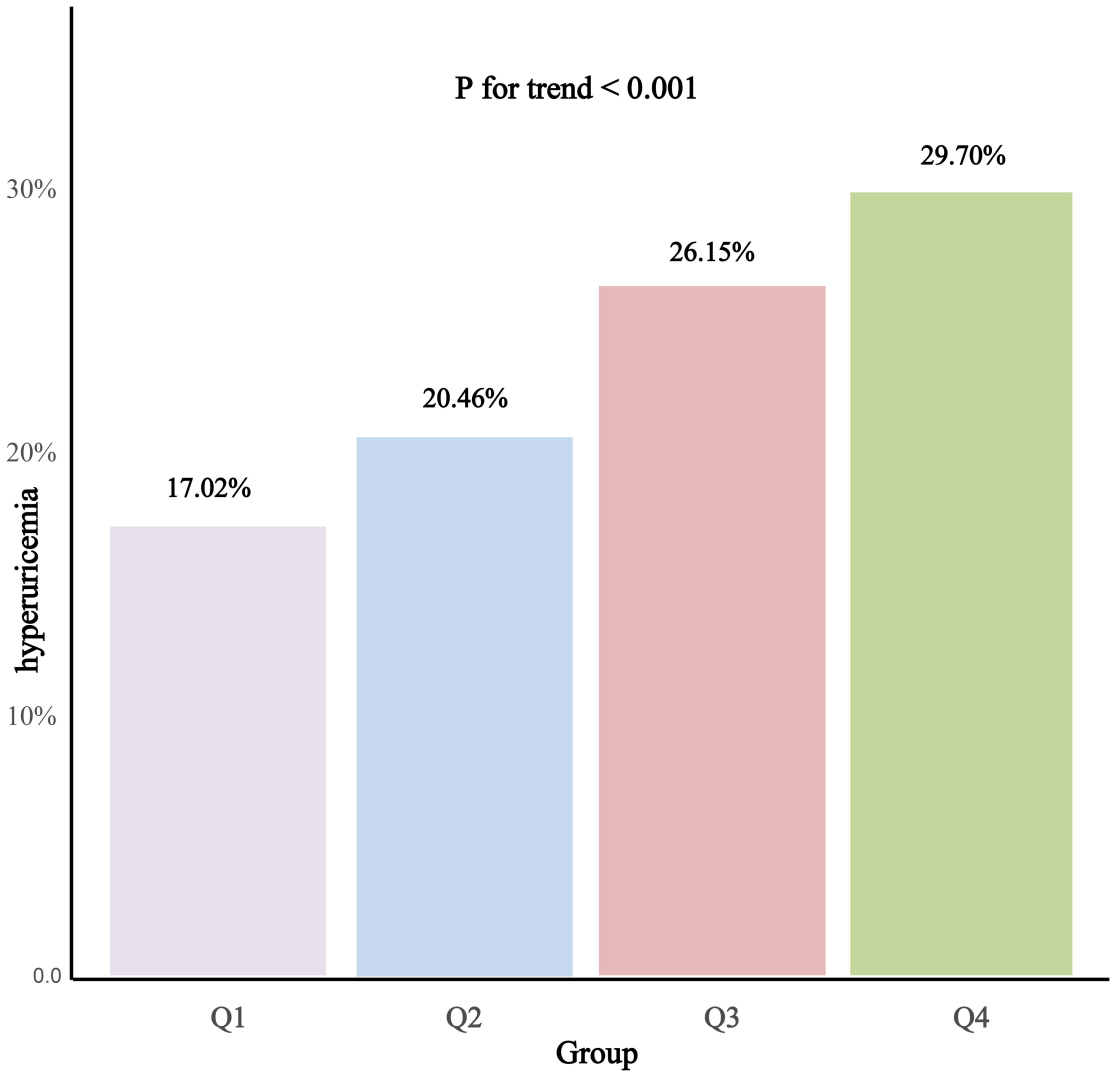
Prevalence of hyperuricemia according to the PAC quartile.

**Figure 3:**
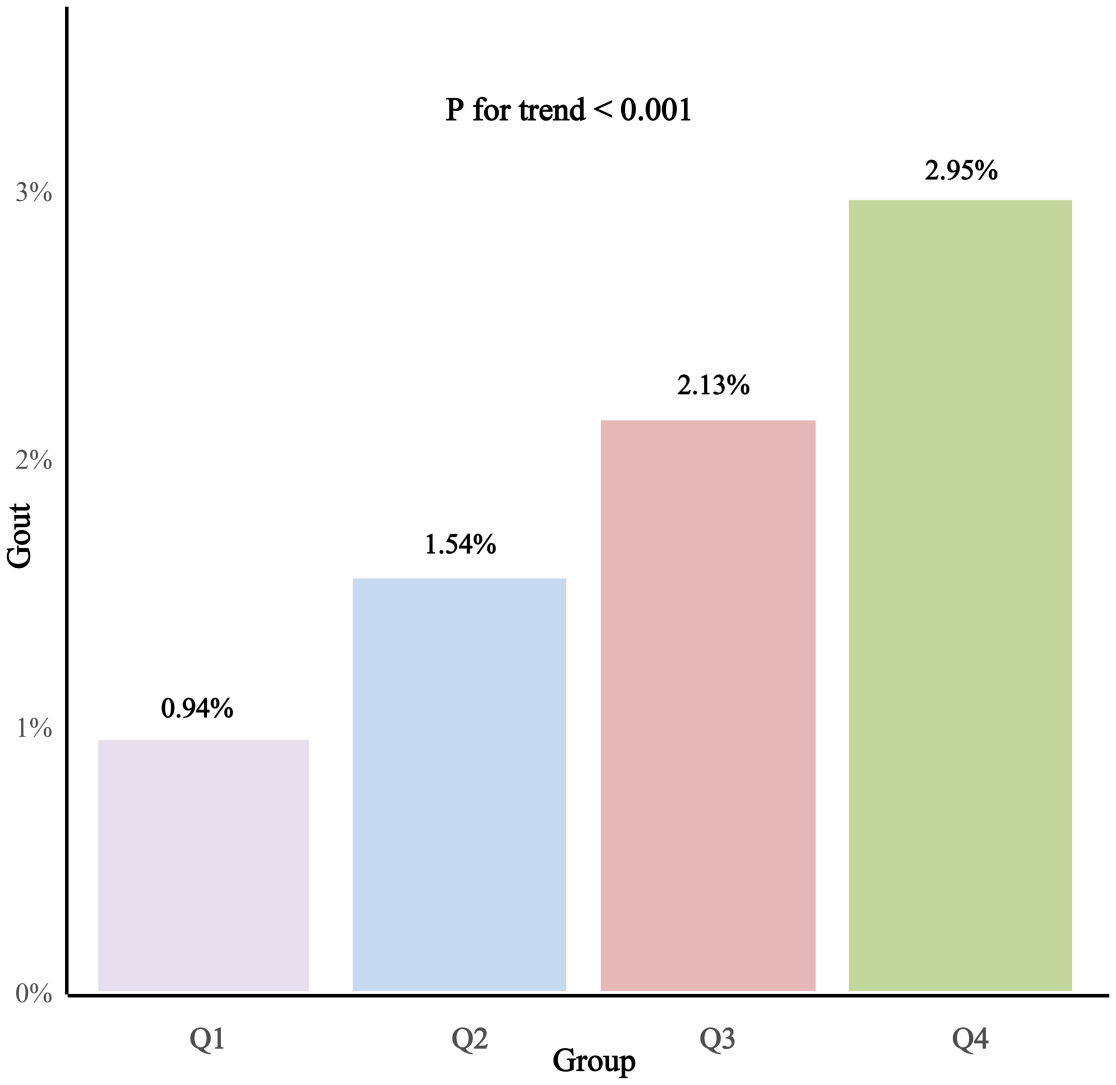
Prevalence of gout according to the PAC quartile.

Laboratory assessments disclosed that Cr, eGFR, LDL-C, and UA levels were significantly elevated in the group with higher PAC levels. Furthermore, an escalation in PAC levels was correlated with an increased prevalence of primary aldosteronism (PA), cancer, and a higher frequency of diuretic and calcium channel blocker usage among patients. Most notably, a significant positive trend was observed, indicating that elevated PAC levels were associated with a higher incidence of hyperuricemia and gout, as illustrated in **Figures 2** and **3**.

Lastly, upon further stratification of participants based on the presence or absence of hyperuricemia, the comparative analysis between these two groups demonstrated largely consistent differences, as detailed in **Supplementary Table S3**.

### 3.2 Relationship between PAC levels and hyperuricemia

**Figure 4:**
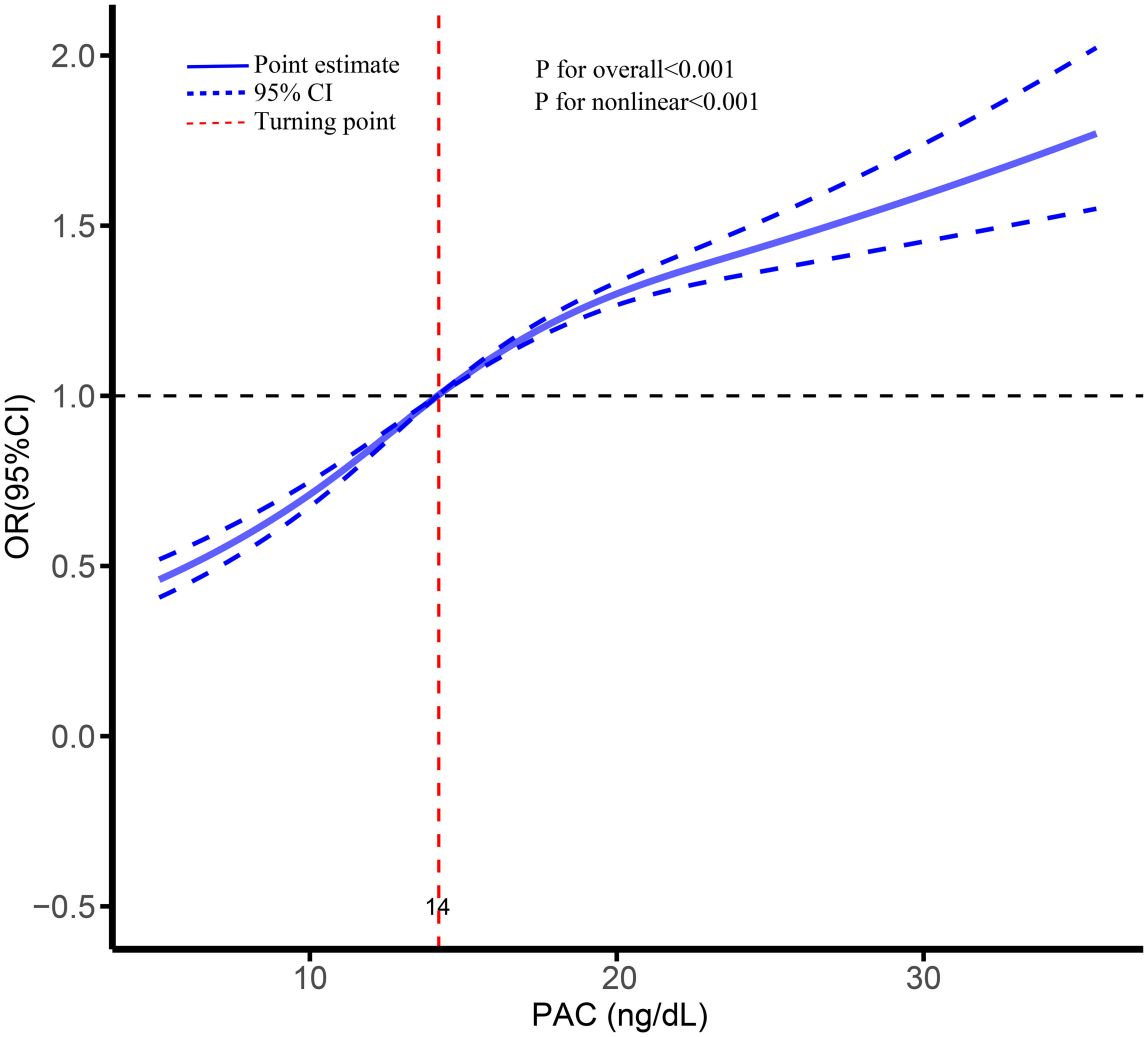
Dose-response association between PAC and risk of hyperuricemia.

Logistic regression analysis revealed a significant association between hyperuricemia and elevated PAC levels (odds ratio [OR], 1.04; 95% confidence interval [CI], 1.04-1.05). This association remained robust in Model 5 after adjusting for all covariates (OR, 1.04; 95% CI, 1.04-1.05). Furthermore, when UA was transformed into a categorical variable, the observed relationship persisted. Notably, compared to the Q1 group, the ORs for hyperuricemia in the Q2, Q3, and Q4 groups were 1.32 (95% CI, 1.25-1.47), 1.94 (95% CI, 1.80-2.10), and 2.24 (95% CI, 2.08-2.42), respectively (**Table 2**, **Supplementary Figure S2**). Additionally, the RCS analysis demonstrated a clear nonlinear dose-response relationship between PAC and hyperuricemia, particularly evident when PAC exceeded 14 ng/dL, indicating a further escalation in the risk of hyperuricemia (**Figure 4**). Employing the threshold analysis before and after the turning point, individuals with PAC exceeding 14 ng/dL exhibited an OR of 1.81 (95%CI, 1.81-1.92) compared to those with PAC below 14 ng/dL (**Table 2**).

**Table 2:** Relationship between PAC and hyperuricemia.

Exposure	Model 1	Model 2	Model 3	Model 4	Model 5
	OR (95% CI) P	OR (95% CI) P	OR (95% CI) P	OR (95% CI) P	OR (95% CI) P
Hyperuricemia					
PAC (per 1-ng/dL increase)	1.04 (1.04, 1.05) <0.001	1.04 (1.04, 1.05) <0.001	1.05 (1.04, 1.05) <0.001	1.05 (1.04, 1.05) <0.001	1.04 (1.04, 1.05) <0.001
Quartiles of PAC					
Q1	Reference	Reference	Reference	Reference	Reference
Q2	1.25 (1.16, 1.35) <0.001	1.25 (1.16, 1.36) <0.001	1.32 (1.22, 1.44) <0.001	1.32 (1.22, 1.44) <0.001	1.32 (1.25, 1.47) <0.001
Q3	1.73 (1.60, 1.86) <0.001	1.73 (1.61, 1.87) <0.001	1.94 (1.79, 2.11) <0.001	1.95 (1.80, 2.11) <0.001	1.94 (1.80, 2.10) <0.001
Q4	2.06 (1.92, 2.22) <0.001	2.05 (1.91, 2.21) <0.001	2.29 (2.12, 2.48) <0.001	2.30 (2.12, 2.49) <0.001	2.24 (2.08, 2.42) <0.001
P for trend	<0.001	<0.001	<0.001	<0.001	<0.001
Threshold (14 ng/dL)					
<=14 ng/dL	Reference	Reference	Reference	Reference	Reference
>14 ng/dL	1.67 (1.58, 1.75) <0.001	1.66 (1.58, 1.75) <0.001	1.81 (1.71, 1.92) <0.001	1.82 (1.72, 1.92) <0.001	1.81 (1.71, 1.92) <0.001

Model 1: no covariates were adjusted.

Model 2: age, sex, BMI, and smoking status were adjusted.

Model 3: Model 2 plus adjustment for PA, diabetes, CHD, and cancer.

Model 4: Model 3 plus adjustment for ALT, AST, Cr, eGFR, TC, TG, HDL.C, LDL.C, FPG, HbA1c, and UA.

Model 5: Model 4 plus adjustment for use of statins, aspirin, diuretics, beta-blockers, calcium channel blockers, ACEIs/ARBs, and oral hypoglycemic agents.

PAC, plasma aldosterone concentration; OR, odds ratio; CI, confidence interval.

Other abbreviations, see **Table 1**.

### 3.3 Relationship between PAC levels and gout

**Figure 5:**
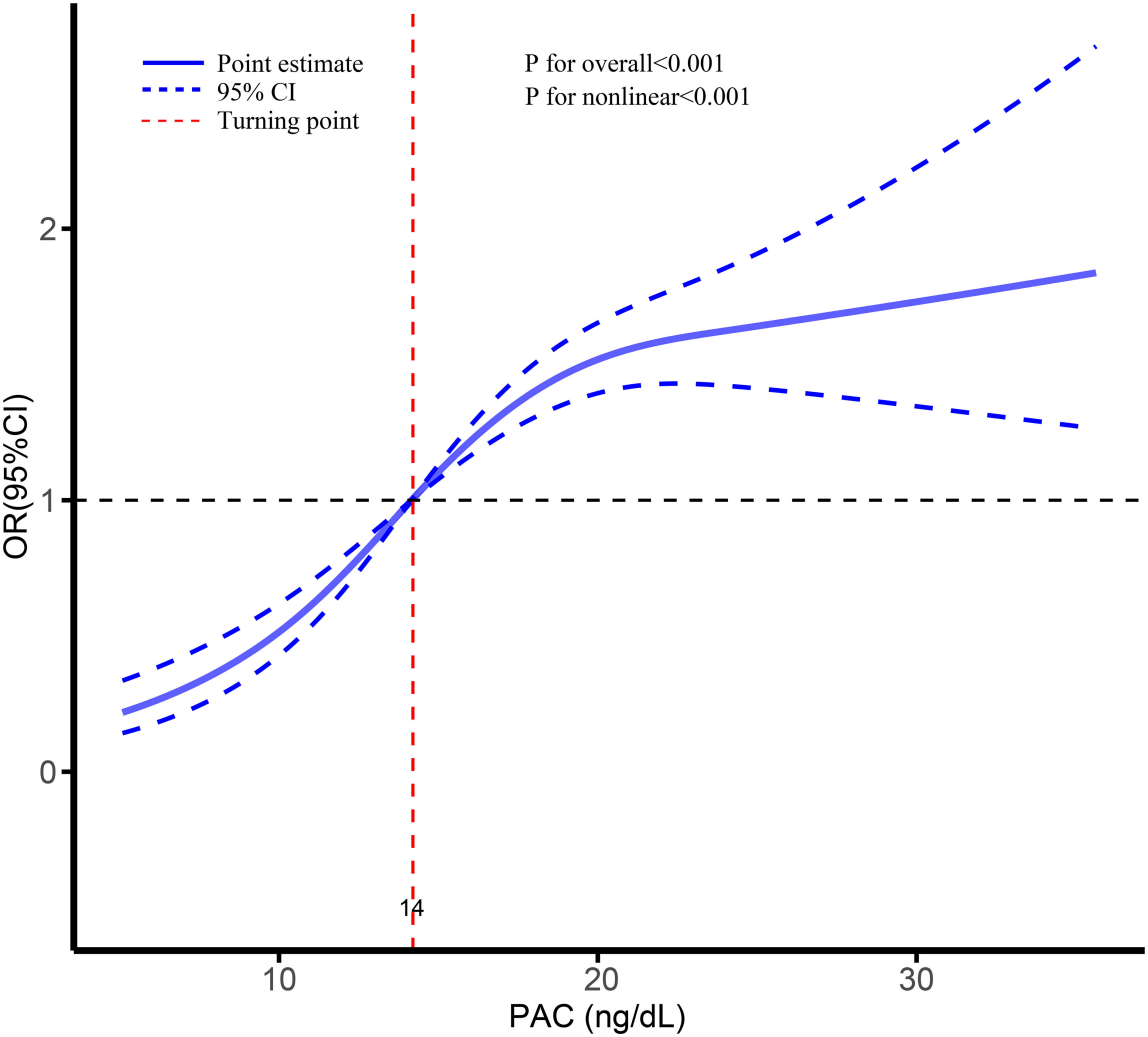
Dose-response association between PAC and risk of gout.

Similar to the relationship observed with hyperuricemia, a clear association was evident between PAC and the risk of gout. After adjusting for various covariates, it was found that for every 1 ng/dL increase in PAC, the risk of developing gout rose by 6%. This trend was further supported by a consistent increase in risk across different PAC quartiles. Specifically, compared to individuals in the Q1 group, those in the Q2, Q3, and highest Q4 groups faced progressively higher odds of developing gout, with ORs of 1.72, 2.43, and 3.23 respectively (**Table 3**, **Supplementary Figure S3**). Moreover, the RCS analysis further underscored this relationship by revealing a significant increase in gout risk when PAC levels exceeded 14 ng/dL (**Figure 5**). Specifically, individuals with PAC levels greater than 14 ng/dL had a 2.14-fold higher risk of gout compared to those with PAC levels below this threshold (**Table 3**).

**Table 3:** Relationship between PAC and gout.

Exposure	Model 1	Model 2	Model 3	Model 4	Model 5
	OR (95% CI) P	OR (95% CI) P	OR (95% CI) P	OR (95% CI) P	OR (95% CI) P
Gout					
PAC (per 1-ng/dL increase)	1.04 (1.04, 1.05) <0.001	1.04 (1.04, 1.05) <0.001	1.05 (1.04, 1.05) <0.001	1.06 (1.04, 1.07) <0.001	1.06 (1.04, 1.07) <0.001
Quartiles of PAC					
Q1	Reference	Reference	Reference	Reference	Reference
Q2	1.65 (1.25, 2.19) <0.001	1.64 (1.25, 2.18) <0.001	1.71 (1.29, 2.27) <0.001	1.71 (1.29, 2.28) <0.001	1.72 (1.30, 2.28) <0.001
Q3	2.30 (1.77, 3.01) <0.001	2.27 (1.75, 2.97) <0.001	2.42 (1.86, 3.18) <0.001	2.43 (1.87, 3.19) <0.001	2.43 (1.87, 3.20) <0.001
Q4	3.21 (2.51, 4.16) <0.001	3.11 (2.42, 4.02) <0.001	3.22 (2.50, 4.18) <0.001	3.23 (2.51, 4.20) <0.001	3.23 (2.51, 4.20) <0.001
P for trend	<0.001	<0.001	<0.001	<0.001	<0.001
Threshold (14 ng/dL)					
<=14 ng/dL	Reference	Reference	Reference	Reference	Reference
>14 ng/dL	2.12 (1.80, 2.51) <0.001	2.07 (1.76, 2.46) <0.001	2.14 (1.81, 2.54) <0.001	2.14 (1.81, 2.55) <0.001	2.14 (1.81, 2.55) <0.001

Model 1: no covariates were adjusted.

Model 2: age, sex, BMI, and smoking status were adjusted.

Model 3: Model 2 plus adjustment for PA, diabetes, CHD, and cancer.

Model 4: Model 3 plus adjustment for ALT, AST, Cr, eGFR, TC, TG, HDL.C, LDL.C, FPG, HbA1c, and UA.

Model 5: Model 4 plus adjustment for use of statins, aspirin, diuretics, beta-blockers, calcium channel blockers, ACEIs/ARBs, and oral hypoglycemic agents.

PAC, plasma aldosterone concentration; OR, odds ratio; CI, confidence interval.

Other abbreviations, see **Table 1**.

### 3.4 Subgroup

To further investigate the influence of PAC across various demographic and health-related stratification factors, an initial categorization of patients was conducted based on their baseline characteristics and health status. This methodological approach produced results that were consistent with the overarching trend observed in the study. It is of particular importance to highlight that the risk for both hyperuricemia and gout increases in tandem with elevated PAC levels, as demonstrated in **Tables 4** and **5**.

**Table 4:** Association between PAC and hyperuricemia in various subgroups.

Variable	Count	Percent	OR 95%CI	P value	P for interaction
**Sex**					<0.001
Female	14889	43.1	1.01 (1.00,1.02)	0.001	
Male	19654	56.9	1.07 (1.06,1.07)	<0.001	
**Age (years)**					0.507
<60	26313	76.2	1.04 (1.04,1.05)	<0.001	
>=60	8230	23.8	1.04 (1.03,1.05)	<0.001	
**BMI (kg/m^2^)**					0.158
<24	7257	21.0	1.05 (1.04,1.06)	<0.001	
>=24	27286	79.0	1.04 (1.04,1.05)	<0.001	
**eGFR (ml/min/1.73 m^2^)**					0.001
<90	5224	15.1	1.03 (1.01,1.04)	<0.001	
>=90	29319	84.9	1.05 (1.04,1.05)	<0.001	
**Current smoking**					<0.001
No	23050	66.7	1.03 (1.03,1.04)	<0.001	
Yes	11493	33.3	1.07 (1.06,1.08)	<0.001	
**CHD**					0.243
No	31361	90.8	1.04 (1.04,1.05)	<0.001	
Yes	3182	9.2	1.05 (1.04,1.07)	<0.001	
**Diabetes**					0.089
No	29013	84.0	1.04 (1.04,1.05)	<0.001	
Yes	5530	16.0	1.05 (1.04,1.06)	<0.001	

Age, sex, BMI, smoking status, PA, diabetes, CHD, cancer, ALT, AST, Cr, eGFR, TC, TG, HDL.C, LDL.C, FPG, HbA1c, UA, statins, aspirin, diuretics, beta-blockers, calcium channel blockers, ACEIs/ARBs, and oral hypoglycemic agents were adjusted.

PAC, plasma aldosterone concentration; OR, odds ratio; CI, confidence interval.

Other abbreviations, see **Table 1**.

**Table 5:** Association between PAC and gout in various subgroups.

Variable	Count	Percent	OR 95%CI	P value	P for interaction
**Sex**					0.004
Female	14889	43.1	1.04 (1.02,1.06)	<0.001	
Male	19654	56.9	1.08 (1.06,1.09)	<0.001	
**Age (years)**					0.951
<60	26313	76.2	1.06 (1.04,1.07)	<0.001	
>=60	8230	23.8	1.06 (1.03,1.09)	<0.001	
**BMI (kg/m^2^)**					0.427
<24	7257	21.0	1.04 (0.99,1.09)	0.080	
>=24	27286	79.0	1.06 (1.05,1.07)	<0.001	
**eGFR (ml/min/1.73 m^2^)**					0.399
<90	5224	15.1	1.04 (1.01,1.08)	0.010	
>=90	29319	84.9	1.06 (1.05,1.07)	<0.001	
**Current smoking**					0.048
No	23050	66.7	1.05 (1.03,1.06)	<0.001	
Yes	11493	33.3	1.08 (1.05,1.10)	<0.001	
**CHD**					0.587
No	31361	90.8	1.06 (1.02,1.06)	<0.001	
Yes	3182	9.2.0	1.05 (1.02,1.06)	0.026	
**Diabetes**					0.096
No	29013	84.0	1.05 (1.02,1.06)	<0.001	
Yes	5530	16.0	1.08 (1.02,1.06)	<0.001	

Age, sex, BMI, smoking status, PA, diabetes, CHD, cancer, ALT, AST, Cr, eGFR, TC, TG, HDL.C, LDL.C, FPG, HbA1c, UA, statins, aspirin, diuretics, beta-blockers, calcium channel blockers, ACEIs/ARBs, and oral hypoglycemic agents were adjusted.

PAC, plasma aldosterone concentration; OR, odds ratio; CI, confidence interval.

Other abbreviations, see **Table 1**.

Subsequently, the analysis was further refined to scrutinize the potential impact of different medications on the study outcomes. The results from this stratified analysis, as outlined in **Supplementary Tables S4** and **S5**, were found to be largely congruent with our initial findings.

Finally, considering the relationship between PAC and hyperuricemia found in this study, when further stratifying by the presence of hyperuricemia, this study also revealed a significantly higher prevalence of PA in individuals with hyperuricemia (**Supplementary Figure S4**).

This concordance serves to underscore the robustness and generalizability of PAC’s effect on UA-related conditions, namely hyperuricemia, and gout, across a spectrum of subgroups.

### 3.5 Sensitivity analysis

In our sensitivity analysis, we first addressed potential biases by excluding missing data from our dataset, which confirmed the stability of the PAC association with both diseases (**Supplementary Tables S6**, **S7**). Furthermore, by removing outliers, we further validated our findings (**Supplementary Tables S8**, **S9**). To mitigate the potential confounding effect of obesity, participants with a BMI > 30 kg/m^2^ were also excluded, ensuring the reliability of our results (**Supplementary Tables S10**, **S11**). Additionally, given that PA patients typically exhibit elevated PAC levels, which might skew the study outcomes, we excluded this demographic to maintain consistency in our analysis (**Supplementary Tables S12**, **S13**). Following this, given the impact of antineoplastic drugs on the study results, we excluded participants with all cancers, and the results were not altered (**Supplementary Tables S14**, **S15**). Of course, considering that diuretic administration may also have some effect on UA metabolism, we further excluded participants taking diuretics, and the results remained robust (**Supplementary Tables S16**, **S17**). Finally, our evaluation of the E value suggests that unmeasured confounding factors have little impact on our results (**Supplementary Tables S18**, **S19**, **Supplementary Figures S5**, **S6**). These analyses further strengthen our conclusion that PAC levels consistently predict the risk of hyperuricemia and gout, regardless of these variables.

## 4 Discussion

Our study marks a pioneering effort to uncover the relationship between PAC and UA in hypertensive patients, demonstrating a significant positive link between aldosterone levels and the occurrence of hyperuricemia and gout. This discovery addresses a previously unexplored gap in the research. The association proved to be stable even after we adjusted for various confounding factors and performed rigorous sensitivity analyses, which attests to the reliability of our findings. Notably, we discovered that when PAC levels exceed 14 ng/dL, the risk for both hyperuricemia and gout increases further, marking this as a pioneering finding in the field. These insights suggest that maintaining PAC levels within a reasonable range may significantly reduce the risk of UA-related diseases in hypertensive patients. Consequently, our study not only fills an important gap in the existing literature but also may offer a novel perspective for future preventive measures and the development of new treatments for hyperuricemia and gout in this population.

UA is a critical metabolite whose long-term dysregulation can lead to not only hyperuricemia and gout but also an increased risk of cardiovascular diseases, including hypertension (5, 29–31). A five-year longitudinal study demonstrated that sustained hyperuricemia elevates the risk of developing hypertension in the future, establishing UA as a significant risk indicator for this condition (31). Moreover, hyperuricemia has been identified as a key risk factor for adolescents with prehypertension and new-onset primary hypertension (32). Early intervention with uric acid-lowering medications is shown to mitigate the risk of hypertension (33). Gout, characterized by joint inflammation, can cause joint deformation and fractures, severely impacting quality of life (29). Thus, preventing hyperuricemia and gout is of paramount importance.

Aldosterone, a crucial element of the RAAS, has been linked to increased blood pressure and damage to various target organs when secreted in excess (21, 22, 34). Although aldosterone and UA are recognized as important markers of hypertension and organ damage, their relationship with UA-related diseases remains underexplored. Some studies have hinted at a potential connection between RAAS activation and UA metabolism, yet this area is contentious (16, 19, 35–38). Research in patients with AF revealed a positive correlation between PAC and UA, suggesting an influence on the progression of UA metabolic diseases (16). Animal studies have further shown that hyperuricemia can result from the activation of mineralocorticoid receptors via mineralocorticoid and glucocorticoid pathways, a process that can be moderated by mineralocorticoid receptor blockers (35, 36). In patients with essential hypertension, long-term use of the aldosterone receptor antagonist spironolactone was found to reduce UA levels (37). Conversely, Mule G et al. reported only a weak link between PAC and UA in untreated essential hypertension, a connection that became statistically insignificant after adjusting for various factors, likely due to the study’s small size and limited population (19). Most prior research has been constrained by small sample sizes and a narrow focus, predominantly in basic research. Our study addresses these gaps with a large participant base, elucidating the association between PAC and UA, their impact on UA-related diseases, and identifying the precise threshold at which PAC levels heighten the risk of hyperuricemia and gout.

The specific mechanism of hyperuricemia and gout caused by aldosterone-induced UA metabolism disorder remains unclear. However, several potential pathways may be involved. Firstly, excessive secretion of aldosterone, a crucial salt corticosteroid, can lead to kidney damage. This damage, in turn, hinders UA excretion, causing UA accumulation and establishing a detrimental cycle of hyperuricemia and kidney impairment (39–42). Secondly, aldosterone might induce inflammation, triggering various inflammatory markers like leukocyte hormones, tumor necrosis factor, and interferon, which disrupt UA metabolism (43–46). Studies on animals have shown that curbing inflammation can mitigate excessive UA secretion (47). Moreover, the abundance of inflammatory secretions can harm blood vessels and kidneys, impacting UA excretion to some extent (48–50). Thirdly, oxidative stress plays a role in this process. Research indicates that aldosterone prompts oxidative stress, activating reactive oxygen species and transcription factors that harm the kidneys (51, 52). Simultaneously, oxidative stress can boost the activity of enzymes in the UA metabolic pathway, directly influencing UA metabolism (45, 53). Finally, aldosterone’s direct impact on UA metabolism is crucial. Studies have demonstrated that aldosterone can impede UA excretion by renal tubules, resulting in UA accumulation and elevated levels in the body (54, 55).

To our knowledge, this is the inaugural large-scale investigation into the correlation between PAC levels and UA-related diseases in hypertensive patients. It is the first to unveil that PAC levels exceeding 14 ng/dL significantly increase the risk of hyperuricemia and gout, providing pivotal insights for the resolution of future disagreements and the enhancement of clinical treatments. The study is characterized by its large sample size and comprehensive clinical data, along with detailed multivariate adjustments and extensive statistical analyses, ensuring the stability of our findings. Nonetheless, interpreting these findings comes with caveats due to several inherent limitations. Firstly, the observational nature of this study precludes the definitive establishment of a causal relationship, which may need to be further confirmed by additional prospective studies in the future. Secondly, our analysis does not account for factors like social status, physical activity, and dietary habits, which affect UA levels, thus limiting the scope of our adjustments. Future studies incorporating detailed dietary assessments could provide further insights into the relationship between PAC and UA-related diseases. Thirdly, the study’s exclusive focus on participants from Northern China suggests caution should be exercised before generalizing these findings. Lastly, despite constructing multiple models to account for a wide array of variables, there remains the possibility of unadjusted confounding factors influencing the results. However, the analysis of the E value shows that there is little chance that our results will be overturned.

## 5 Conclusion

This study is the first to explore the relationship between PAC and UA-related diseases like hyperuricemia and gout in hypertensive patients. It is pioneering to find that when PAC levels exceed 14 ng/dL, the risk of hyperuricemia and gout increases significantly. This discovery could have vital clinical implications for the early prevention and treatment of hyperuricemia and gout in hypertensive patients. However, further prospective studies are necessary to validate these findings.

## Data availability statement

The original contributions presented in the study are included in the article/**Supplementary Material**. Further inquiries can be directed to the corresponding author.

## Ethics statement

This study was approved by the Research Ethics Committee of the Xinjiang Uygur Autonomous Region People’s Hospital (KY2022080905). All the procedures complied with the requirements of the Declaration of Helsinki. All participants provided informed written consent. The studies were conducted in accordance with the local legislation and institutional requirements. Written informed consent for participation was not required from the participants or the participants’ legal guardians/next of kin in accordance with the national legislation and institutional requirements.

## Author contributions

SS: Conceptualization, Data curation, Formal Analysis, Investigation, Methodology, Software, Validation, Writing – original draft, Writing – review & editing. XC: Conceptualization, Data curation, Formal Analysis, Investigation, Methodology, Software, Writing – original draft, Writing – review & editing. JHu: Conceptualization, Formal Analysis, Funding acquisition, Investigation, Methodology, Writing – review & editing. QZ: Data curation, Formal Analysis, Resources, Writing – review & editing. DS: Methodology, Software, Writing – review & editing. HM: Methodology, Writing – review & editing. YZ: Data curation, Writing – review & editing. RM: Data curation, Writing – review & editing. PZ: Data curation, Writing – review & editing. WY: Methodology, Writing – review & editing. JHo: Project administration, Supervision, Writing – review & editing. DZ: Funding acquisition, Project administration, Writing – review & editing. NL: Conceptualization, Data curation, Methodology, Project administration, Resources, Supervision, Validation, Writing – review & editing.
