# Supplementary material for: Plasma aldosterone concentrations elevation in hypertensive patients: the dual impact on hyperuricemia and gout
Source: Front Endocrinol (Lausanne). 2024 Jul 30;15:1424207. doi: 10.3389/fendo.2024.1424207 (PMC11319118; doi:10.3389/fendo.2024.1424207)
Supplement: Supplementary file 1 [file DataSheet_1.docx]

Supplementary Material

# Supplemental material and methods

**Baseline examination**

Anthropometric measurements were taken by trained nurses. Data for height and weight were acquired following a protocol standardized to an accuracy of 0.1 kg and 0.1 cm, respectively. Current smokers were defined as having smoked 100 cigarettes in their lifetime and currently smoking. Blood pressure was measured using a mercury sphygmomanometer after the patient had rested quietly for at least 10 minutes, and the average of multiple measurements was taken as the systolic and diastolic blood pressure values. All biochemical tests were measured by blood sampling after an overnight fast.

**Definitions**

Criteria for hypertension included self-reported hypertension, current use of anti-hypertensive medication, or systolic blood pressure (SBP) ≥ 140 mmHg and/or diastolic blood pressure (DBP) ≥ 90 mmHg recorded for at least three consecutive readings. Diabetes was defined as fasting serum glucose ≥7.0 mmol/L, the 2-hour serum glucose of the oral glucose tolerance test ≥11.1 mmol/L, or the current use of hypoglycemic medication or insulin. Coronary heart disease (CHD) was defined as a fatal or nonfatal myocardial infarction, unstable angina, and coronary revascularization. The diagnosis of diabetes is determined by several criteria, including the past and current use of any hypoglycemic drug, a fasting blood glucose level greater than 7.0 mmol/L, a 2-hour oral glucose tolerance test blood glucose level greater than or equal to 11.1 mmol/L, or a random blood glucose level greater than or equal to 11.1 mmol/L. Diabetes is diagnosed if any of these criteria are met (1). To identify primary aldosteronism (PA) according to the Endocrine Society's Clinical Practice Guidelines. For the measurement of plasma aldosterone concentrations (PAC), participants were asked to sit for 30 minutes after being active for at least 2 hours before blood collection, which took place between 8:00 and 11:00 AM. PAC was measured using radio-immunoassay (DSL-8600 ACTIVE Aldosterone Coated Tube Radioimmunoassay Kit; Diagnostic Systems Laboratories, Webster, TX). Plasma renin activity was also measured by radioimmunoassay using commercial kits (Center of Beifang Biology Technique, Beijing, China). The plasma samples were divided into 2 parts: one for the determination of plasma AIa (angiotensin I) concentrations after reacting the sample with direct antibodies, and the other for the determination of plasma AIb concentrations following a 1-hour incubation at 37 °C and then reacting the sample with direct antibodies (2, 3). Patients were diagnosed with PA based on a PAC level of ≥ 12 ng/dL, an aldosterone-to-renin ratio ≥ 20, and a PAC value ≥ 10 ng/dL confirmed by saline infusion testing (4). The body mass index (BMI) was calculated as per the formula: weight (kg)/height^2^ (m).

**Details of the statistical analyses**.

Considering that there are missing values in a part of the study data, in order to solve this problem, we generated five sets of interpolated data by multiple interpolation using the packages of mice in R. The data sets were then combined to form a complete data set. The statistical values of these five sets of data were then combined to form a complete data set for the final analysis. The interpolated data showed essentially the same trend as the original data, which proved the reliability of the interpolation method.

Variables of baseline characteristics are shown as n (%) if categorical, mean (SD) if normally distributed, and median (interquartile range) if nonnormally distributed. In order to compare features between the two groups, the chi-square test was used for categorical variables, and one-way analysis of variance, or the Kruskal-Wallis test, was used for continuous variables with normal and skew distributions.

Prior to any regression analysis, we tested multicollinearity among the predictors by checking for the variance inflation factor. Multicollinearity analyses revealed that variance inflation factors were smaller than 10 for all predictor variables, confirming that regression models were not affected by the presence of multicollinearity. The relationship between PAC and hyperuricemia and gout was examined by multivariate logistic regression model. This study set five different models (Model 1: no covariates were adjusted; Model 2: adjusted for age, sex, BMI, and smoking status; Model 3: Model 2 plus adjustment for PA, DM, CHD, and cancer; Model 4: Model 3 plus adjustment for ALT, AST, Cr, eGFR, TC, TG, HDL.C, LDL.C, FPG, HbA1c, and UA; Model 5: Model 4 plus adjustment for use of statins, aspirin, diuretics, beta-blockers, calcium channel blockers, ACEIs/ARBs, and oral hypoglycemic agents. In addition, the restricted inverse square spline (four nodes at the 5th, 35th, 65th, and 95th percentiles of the PAC) was used to evaluate the dose-response relationship between PAC and the risks of hyperuricemia and gout, and the turning points were further calculated using a recursive algorithm. Based on the turning points of the RCS, we can further look for a threshold effect between the level of PAC and the study outcome, which may provide a fuller theoretical basis for guiding clinical work. Moreover, based on the turning point, we conducted threshold analysis before and after the turning point. In addition, subgroup analyses were conducted by gender, age, BMI, eGFR, current smoking, CHD, diabetes, and use of various medication types, and multiplicative interaction tests were applied.

We performed a series of sensitivity analyses to assess the robustness of our findings as follows: First, we excluded data with missing values to judge consistency with the overall trend. Second, in order to verify the reliability of the results, we analyzed the relationship between PAC and hyperuricemia and gout after excluding outliers (PAC less than -3SD or more than +3SD). Third, we again excluded excessively obese participants with BMI > 30 kg/m^2^. Following this, we also excluded patients with PA to verify the reliability of our results. Following this, given the impact of antineoplastic drugs on the study results, we excluded patients with serious illnesses such as cancer, and this result was still unaffected. Of course, considering that diuretic administration may also have some effect on uric acid metabolism, for this reason we further excluded participants taking diuretics, and the results remained robust. Finally, our assessment of the E-values indicated that the impact of unmeasured confounders on our results was minimal, reinforcing the overall reliability of our findings.

All analyses were done using R (4.2.2). All P-values were two-sided, and P-values of <0.05 denoted statistical significance.

**2 Supplementary Tables**

**Table S1**. List of medications included in the study

| Drug class | Drug name |
| --- | --- |
| Aspirin | Aspirin |
| Statin | Atorvastatin, fluvastatin, pitavastatin, rosuvastatin, simvastatin |
| Diuretics | Acetazolamide, amiloride, benzyl hydrochlorothiazide, bumetanide, furosemide, hydrochlorothiazide, indapamide, spironolactone |
| Beta-blocker | Atenolol, bisoprolol, carvedilol, metoprolol, propranolol |
| Calcium channel blockers | Amlodipine, diltiazem, felodipine, lercanidipine, nifedipine,  verapamil |
| Angiotensin-converting enzyme inhibitors or angiotensin receptor blockers | Azilsartan, candestartan, captopril, enalapril, fosinopril, irbesartan, losartan, olmesartan, ramipril telmisartan, valsartan |
| Oral antidiabetic agents | Metformin, glipizide, gliclazide, glimepiride, glyburide, alogliptin, linagliptin, sitagliptin, vidagliptin, saxagliptin, acarbose, nateglinide, meglitinide, repaglinde, pioglitzone,dulaglutide, exenatide, liraglutide |

**Table S2**. Covariance Diagnostics

| **Variable** | **VIF** |
| --- | --- |
| Age | 3.146775 |
| Sex | 2.002386 |
| BMI | 1.128773 |
| SBP | 1.740032 |
| DBP | 1.816712 |
| Current smoking | 1.573644 |
| ALT | 3.456508 |
| AST | 3.217181 |
| TC | 1.424347 |
| TG | 1.527557 |
| HDL.C | 1.589715 |
| LDL.C | 1.004766 |
| Cr | 1.256333 |
| BUN | 1.162688 |
| eGFR | 3.560288 |
| FPG | 2.474721 |
| HbA1c | 2.451157 |
| UA | 1.127536 |
| PA | 1.006599 |
| DM | 2.728855 |
| CHD | 1.18911 |
| Statins | 1.151215 |
| Diuretics | 1.164649 |
| Beta-blockers | 1.111626 |
| Calcium channel blockers | 1.147902 |
| ACEIs/ARBs | 1.254601 |
| Oral hypoglycemic agents | 1.69706 |

VIF = 1/(1-R^2^). VIF step-by-step screening method: Calculate the VIF of each variable. If the maximum VIF value is ≥ 10, remove the variable with the maximum VIF value.

VIF: variance inflation factors. For other abbreviations, see Table 1.

**Table S3.** Characteristics of the study population based on with or without hyperuricemia

| Characteristic | Overall | Non-hyperuricemia | Hyperuricemia | P value |
| --- | --- | --- | --- | --- |
| N | 34543 | 26483 | 8060 |  |
| Age (years) | 51.10±12.41 | 51.36±12.44 | 50.25±12.27 | <0.001 |
| BMI (kg/m^2^) | 26.92±3.63 | 26.58±3.63 | 28.03±3.42 | <0.001 |
| Sex |  |  |  | <0.001 |
| Female | 14889 (43.10%) | 11282 (42.60%) | 3607 (44.75%) |  |
| Male | 19654 (56.90%) | 15201 (57.40%) | 4453 (55.25%) |  |
| SBP (mmHg) | 146.01±18.27 | 145.71±18.32 | 147.02±18.07 | <0.001 |
| DBP (mmHg) | 88.10±13.59 | 87.22±13.46 | 90.99±13.63 | <0.001 |
| Current smoking (%) | 11493 (33.27%) | 8870 (33.49%) | 2623 (32.54%) | 0.113 |
| **Medical history** |  |  |  |  |
| PA (%) | 5209 (15.08%) | 3997 (15.09%) | 1212 (15.04%) | 0.903 |
| DM (%) | 5530 (16.01%) | 4182 (15.79%) | 1348 (16.72%) | 0.045 |
| CHD (%) | 3182 (9.21%) | 2438 (9.21%) | 744 (9.23%) | 0.946 |
| Cancer (%) | 504 (1.46%) | 378 (1.43%) | 126 (1.56%) | 0.612 |
| **Laboratory tests** |  |  |  |  |
| ALT (U/L) | 27.02±17.48 | 25.14±16.27 | 33.20±19.76 | <0.001 |
| AST (U/L) | 20.91±8.12 | 20.35±7.75 | 22.78±8.99 | <0.001 |
| Cr (umol/L) | 65.13±14.63 | 62.79±13.97 | 72.83±14.12 | <0.001 |
| eGFR (ml/min/1.73 m^2^) | 115.90±27.18 | 115.57±27.12 | 117.00±27.37 | <0.001 |
| BUN (mmol/L) | 5.05±1.35 | 4.98±1.34 | 5.28±1.37 | <0.001 |
| TC (mmol/L) | 4.51±0.95 | 4.48±0.95 | 4.63±0.95 | <0.001 |
| TG (mmol/L) | 1.74±0.90 | 1.63±0.83 | 2.11±0.99 | <0.001 |
| HDL.C (mg/dL) | 1.06±0.25 | 1.08±0.25 | 0.98±0.22 | <0.001 |
| LDL.C (mg/dL) | 2.75±0.82 | 2.75±0.82 | 2.76±0.83 | 0.227 |
| FPG (mmol/L) | 5.05±1.10 | 5.04±1.10 | 5.07±1.12 | 0.012 |
| HbA1c (%) | 5.94±0.84 | 5.93±0.84 | 5.97±0.85 | <0.001 |
| UA (umol/L) | 307.43±108.35 | 261.59±72.76 | 458.05±57.70 | <0.001 |
| PAC (ng/dL) | 15.80±5.63 | 15.47±5.51 | 16.87±5.88 | <0.001 |
| **Medications** |  |  |  |  |
| Statins (%) | 3937 (11.40%) | 2985 (11.27%) | 952 (11.81%) | 0.182 |
| Aspirins (%) | 3972 (11.50%) | 3022 (11.41%) | 950 (11.79%) | 0.355 |
| Diuretics (%) | 3713 (10.75%) | 2763 (10.43%) | 950 (11.79%) | <0.001 |
| Beta-blockers (%) | 6116 (17.71%) | 4636 (17.51%) | 1480 (18.36%) | 0.078 |
| Calcium channel blockers (%) | 18224 (52.76%) | 13891 (52.45%) | 4333 (53.76%) | 0.04 |
| ACEIs/ARBs (%) | 15925 (46.10%) | 12123 (45.78%) | 3802 (47.17%) | 0.028 |
| Oral hypoglycemic agents (%) | 2721 (7.88%) | 2111 (7.97%) | 610 (7.57%) | 0.24 |

Data are presented as mean ± standard deviation, or as numbers, and percentages.

Abbreviations: BMI, body mass index; SBP, systolic blood pressure; DBP, diastolic blood pressure; PA, primary aldosteronism; CHD, coronary heart disease; ALT, alanine transaminase; AST, aspartate transaminase; Cr, creatinine; eGFR, estimated glomerular filtration rate; BUN, blood urea nitrogen; TC, total cholesterol; TG, triglyceride; HDL-C, high-density lipoprotein cholesterol; LDL-C, low-density lipoprotein cholesterol; FPG, fasting plasma glucose; HbA1c, glycosylated hemoglobin; UA, Uric acid; PAC, plasma aldosterone concentration; ARBs, angiotensin receptor blockers; ACEIs, angiotensin-converting enzyme inhibitors.

**Table S4.** Association between PAC and hyperuricemia in medication subgroups

| Variable | Count | Percent | OR 95%CI | P value | P for interaction |
| --- | --- | --- | --- | --- | --- |
| **Statins** |  |  |  |  | 0.869 |
| No | 30606 | 88.6 | 1.04 (1.04,1.05) | <0.001 |  |
| Yes | 3937 | 11.4 | 1.04 (1.03,1.06) | <0.001 |  |
| **Diuretics** |  |  |  |  | 0.668 |
| No | 30830 | 89.3 | 1.04 (1.04,1.05) | <0.001 |  |
| Yes | 3713 | 10.7 | 1.04 (1.03,1.05) | <0.001 |  |
| **Beta-blockers** |  |  |  |  | 0.927 |
| No | 28427 | 82.3 | 1.04 (1.04,1.05) | <0.001 |  |
| Yes | 6116 | 17.7 | 1.04 (1.03,1.05) | <0.001 |  |
| **Calcium channel blockers** |  |  |  |  | 0.249 |
| No | 16319 | 47.2 | 1.05 (1.04,1.05) | <0.001 |  |
| Yes | 18224 | 52.8 | 1.04 (1.03,1.05) | <0.001 |  |
| **ACEIs/ARBs** |  |  |  |  | 0.339 |
| No | 18618 | 53.9 | 1.05 (1.04,1.05) | <0.001 |  |
| Yes | 15925 | 46.1 | 1.04 (1.03,1.05) | <0.001 |  |
| **Oral hypoglycemic agents** |  |  |  |  | 0.213 |
| No | 31822 | 92.1 | 1.04 (1.04,1.05) | <0.001 |  |
| Yes | 2721 | 7.9 | 1.05 (1.04,1.07) | <0.001 |  |

Abbreviations: PAC, plasma aldosterone concentration; OR, odds ratio; CI, confidence interval.

Other abbreviations, see Table 1.

**Table S5.** Association between PAC and gout in medication subgroups

| Variable | Count | Percent | OR 95%CI | P value | P for interaction |
| --- | --- | --- | --- | --- | --- |
| **Statins** |  |  |  |  | 0.639 |
| No | 30606 | 88.6 | 1.06 (1.04,1.07) | <0.001 |  |
| Yes | 3937 | 11.4 | 1.07 (1.03,1.10) | <0.001 |  |
| **Diuretics** |  |  |  |  | 0.850 |
| No | 30830 | 89.3 | 1.06 (1.05,1.07) | <0.001 |  |
| Yes | 3713 | 10.7 | 1.05 (1.02.1.09) | 0.002 |  |
| **Beta-blockers** |  |  |  |  | 0.012 |
| No | 28427 | 82.3 | 1.07 (1.05,1.08) | <0.001 |  |
| Yes | 6116 | 17.7 | 1.02 (1.01,1.05) | 0.021 |  |
| **Calcium channel blockers** |  |  |  |  | 0.270 |
| No | 16319 | 47.2 | 1.07 (1.05,1.09) | <0.001 |  |
| Yes | 18224 | 52.8 | 1.05 (1.03,1.07) | <0.001 |  |
| **ACEIs/ARBs** |  |  |  |  | 0.978 |
| No | 18618 | 53.9 | 1.06 (1.04,1.08) | <0.001 |  |
| Yes | 15925 | 46.1 | 1.06 (1.04,1.08) | <0.001 |  |
| **Oral hypoglycemic agents** |  |  |  |  | 0.055 |
| No | 31822 | 92.1 | 1.05 (1.04,1.07) | <0.001 |  |
| Yes | 2721 | 7.9 | 1.10 (1.05,1.14) | <0.001 |  |

Abbreviations: PAC, plasma aldosterone concentration; OR, odds ratio; CI, confidence interval.

Other abbreviations, see Table 1.

**Table S6.** Sensitivity analyses of the association of PAC with hyperuricemia using a no-missing value dataset

| Exposure | Model 1 | Model 2 | Model 3 | Model 4 | Model 5 |
| --- | --- | --- | --- | --- | --- |
|  | OR (95% CI) P | OR (95% CI) P | OR (95% CI) P | OR (95% CI) P | OR (95% CI) P |
| **Hyperuricemia** |  |  |  |  |  |
| PAC (per 1-ng/dL increase) | 1.04 (1.04, 1.05)  <0.001 | 1.04 (1.04, 1.05)  <0.001 | 1.05 (1.04, 1.05)  <0.001 | 1.05 (1.05, 1.06)  <0.001 | 1.05 (1.05, 1.06)  <0.001 |
| Quartiles of PAC |  |  |  |  |  |
| Q1 | Reference | Reference | Reference | Reference | Reference |
| Q2 | 1.28 (1.19, 1.39)  <0.001 | 1.28 (1.19, 1.39)  <0.001 | 1.36 (1.25, 1.48)  <0.001 | 1.36 (1.25, 1.48)  <0.001 | 1.36 (1.25, 1.48)  <0.001 |
| Q3 | 1.76 (1.64, 1.90)  <0.001 | 1.77 (1.64, 1.91)  <0.001 | 1.99 (1.83, 2.16)  <0.001 | 2.01 (1.84, 2.17)  <0.001 | 1.99 (1.83, 2.17)  <0.001 |
| Q4 | 2.11 (1.96, 2.27)  <0.001 | 2.10 (1.95, 2.27)  <0.001 | 2.36 (2.18, 2.56)  <0.001 | 2.37 (2.19, 2.57)  <0.001 | 2.37 (2.18, 2.57)  <0.001 |
| P for trend | <0.001 | <0.001 | <0.001 | <0.001 | <0.001 |

Model 1: no covariates were adjusted.

Model 2: age, sex, BMI, and smoking status were adjusted.

Model 3: Model 2 plus adjustment for PA, diabetes, CHD, and cancer.

Model 4: Model 3 plus adjustment for ALT, AST, Cr, eGFR, TC, TG, HDL.C, LDL.C, FPG, HbA1c, and UA.

Model 5: Model 4 plus adjustment for use of statins, aspirin, diuretics, beta-blockers, calcium channel blockers, ACEIs/ARBs, and oral hypoglycemic agents.

Abbreviations: PAC, plasma aldosterone concentration; OR, odds ratio; CI, confidence interval.

Other abbreviations, see Table 1.

**Table S7.** Sensitivity analyses of the association of PAC with gout using a no-missing value dataset

| Exposure | Model 1 | Model 2 | Model 3 | Model 4 | Model 5 |
| --- | --- | --- | --- | --- | --- |
|  | OR (95% CI) P | OR (95% CI) P | OR (95% CI) P | OR (95% CI) P | OR (95% CI) P |
| **Gout** |  |  |  |  |  |
| PAC (per 1-ng/dL increase) | 1.06 (1.04, 1.07)  <0.001 | 1.06 (1.04, 1.07)  <0.001 | 1.06 (1.04, 1.07)  <0.001 | 1.06 (1.04, 1.07)  <0.001 | 1.06 (1.04, 1.07)  <0.001 |
| Quartiles of PAC |  |  |  |  |  |
| Q1 | Reference | Reference | Reference | Reference | Reference |
| Q2 | 1.69 (1.28, 2.25)  <0.001 | 1.68 (1.27, 2.23)  <0.001 | 1.75 (1.32, 2.34)  <0.001 | 1.76 (1.32, 2.35)  <0.001 | 1.76 (1.33, 2.35)  <0.001 |
| Q3 | 2.35 (1.81, 3.09)  <0.001 | 2.31 (1.78, 3.04)  <0.001 | 2.48 (1.90, 3.28)  <0.001 | 2.49 (1.90, 3.28)  <0.001 | 2.49 (1.91, 3.29)  <0.001 |
| Q4 | 3.26 (2.53, 4.23)  <0.001 | 3.14 (2.44, 4.09)  <0.001 | 3.28 (2.54, 4.29)  <0.001 | 3.29 (2.55, 4.30)  <0.001 | 3.29 (2.55, 4.30)  <0.001 |
| P for trend | <0.001 | <0.001 | <0.001 | <0.001 | <0.001 |

Model 1: no covariates were adjusted.

Model 2: age, sex, BMI, and smoking status were adjusted.

Model 3: Model 2 plus adjustment for PA, diabetes, CHD, and cancer.

Model 4: Model 3 plus adjustment for ALT, AST, Cr, eGFR, TC, TG, HDL.C, LDL.C, FPG, HbA1c, and UA.

Model 5: Model 4 plus adjustment for use of statins, aspirin, diuretics, beta-blockers, calcium channel blockers, ACEIs/ARBs, and oral hypoglycemic agents.

Abbreviations: PAC, plasma aldosterone concentration; OR, odds ratio; CI, confidence interval.

Other abbreviations, see Table 1.

**Table S8.** Sensitivity analysis of the relationship between PAC with hyperuricemia was performed after excluding outliers

| Exposure | Model 1 | Model 2 | Model 3 | Model 4 | Model 5 |
| --- | --- | --- | --- | --- | --- |
|  | OR (95% CI) P | OR (95% CI) P | OR (95% CI) P | OR (95% CI) P | OR (95% CI) P |
| **Hyperuricemia** |  |  |  |  |  |
| PAC (per 1-ng/dL increase) | 1.05 (1.04, 1.05)  <0.001 | 1.05 (1.04, 1.05)  <0.001 | 1.05 (1.05, 1.06)  <0.001 | 1.05 (1.05, 1.06)  <0.001 | 1.05 (1.05, 1.06)  <0.001 |
| Quartiles of PAC |  |  |  |  |  |
| Q1 | Reference | Reference | Reference | Reference | Reference |
| Q2 | 1.25 (1.16, 1.35)  <0.001 | 1.25 (1.16, 1.36)  <0.001 | 1.32 (1.22, 1.44)  <0.001 | 1.33 (1.22, 1.44)  <0.001 | 1.32 (1.22, 1.44)  <0.001 |
| Q3 | 1.73 (1.60, 1.86)  <0.001 | 1.73 (1.61, 1.87)  <0.001 | 1.94 (1.79, 2.11)  <0.001 | 1.95 (1.80, 2.12)  <0.001 | 1.95 (1.80, 2.11)  <0.001 |
| Q4 | 2.05 (1.91, 2.21)  <0.001 | 2.05 (1.90, 2.20)  <0.001 | 2.28 (2.10, 2.47)  <0.001 | 2.29 (2.11, 2.48)  <0.001 | 2.29 (2.11, 2.48)  <0.001 |
| P for trend | <0.001 | <0.001 | <0.001 | <0.001 | <0.001 |

Values more than 3SD from the mean were deemed as outliers, they were removed and the analysis repeated.

Model 1: no covariates were adjusted.

Model 2: age, sex, BMI, and smoking status were adjusted.

Model 3: Model 2 plus adjustment for PA, diabetes, CHD, and cancer.

Model 4: Model 3 plus adjustment for ALT, AST, Cr, eGFR, TC, TG, HDL.C, LDL.C, FPG, HbA1c, and UA.

Model 5: Model 4 plus adjustment for use of statins, aspirin, diuretics, beta-blockers, calcium channel blockers, ACEIs/ARBs, and oral hypoglycemic agents.

Abbreviations: PAC, plasma aldosterone concentration; OR, odds ratio; CI, confidence interval.

Other abbreviations, see Table 1.

**Table S9.** Sensitivity analysis of the relationship between PAC with gout was performed after excluding outliers

| Exposure | Model 1 | Model 2 | Model 3 | Model 4 | Model 5 |
| --- | --- | --- | --- | --- | --- |
|  | OR (95% CI) P | OR (95% CI) P | OR (95% CI) P | OR (95% CI) P | OR (95% CI) P |
| **Gout** |  |  |  |  |  |
| PAC (per 1-ng/dL increase) | 1.06 (1.05, 1.08)  <0.001 | 1.06 (1.05, 1.08)  <0.001 | 1.06 (1.05, 1.08)  <0.001 | 1.06 (1.05, 1.08)  <0.001 | 1.06 (1.05, 1.08)  <0.001 |
| Quartiles of PAC |  |  |  |  |  |
| Q1 | Reference | Reference | Reference | Reference | Reference |
| Q2 | 1.65 (1.25, 2.19)  <0.001 | 1.64 (1.25, 2.18)  <0.001 | 1.71 (1.29, 2.27)  <0.001 | 1.71 (1.30, 2.28)  <0.001 | 1.72 (1.30, 2.29)  <0.001 |
| Q3 | 2.30 (1.77, 3.01)  <0.001 | 2.27 (1.75, 2.97)  <0.001 | 2.42 (1.86, 3.18)  <0.001 | 2.43 (1.87, 3.19)  <0.001 | 2.44 (1.87, 3.20)  <0.001 |
| Q4 | 3.23 (2.52, 4.18)  <0.001 | 3.12 (2.43, 4.04)  <0.001 | 3.24 (2.51, 4.21)  <0.001 | 3.25 (2.52, 4.23)  <0.001 | 3.22 (2.52, 4.24)  <0.001 |
| P for trend | <0.001 | <0.001 | <0.001 | <0.001 | <0.001 |

Values more than 3SD from the mean were deemed as outliers, they were removed and the analysis repeated.

Model 1: no covariates were adjusted.

Model 2: age, sex, BMI, and smoking status were adjusted.

Model 3: Model 2 plus adjustment for PA, diabetes, CHD, and cancer.

Model 4: Model 3 plus adjustment for ALT, AST, Cr, eGFR, TC, TG, HDL.C, LDL.C, FPG, HbA1c, and UA.

Model 5: Model 4 plus adjustment for use of statins, aspirin, diuretics, beta-blockers, calcium channel blockers, ACEIs/ARBs, and oral hypoglycemic agents.

Abbreviations: PAC, plasma aldosterone concentration; OR, odds ratio; CI, confidence interval.

Other abbreviations, see Table 1.

**Table S10.** Sensitivity analysis of the relationship between PAC with hyperuricemia was performed after excluding patients with BMI > 30 kg/m^2^.

| Exposure | Model 1 | Model 2 | Model 3 | Model 4 | Model 5 |
| --- | --- | --- | --- | --- | --- |
|  | OR (95% CI) P | OR (95% CI) P | OR (95% CI) P | OR (95% CI) P | OR (95% CI) P |
| **Hyperuricemia** |  |  |  |  |  |
| PAC (per 1-ng/dL increase) | 1.04 (1.04, 1.05)  <0.001 | 1.04 (1.04, 1.05)  <0.001 | 1.05 (1.04, 1.05)  <0.001 | 1.05 (1.04, 1.05)  <0.001 | 1.05 (1.04, 1.05)  <0.001 |
| Quartiles of PAC |  |  |  |  |  |
| Q1 | Reference | Reference | Reference | Reference | Reference |
| Q2 | 1.27 (1.16, 1.38)  <0.001 | 1.27 (1.16, 1.39)  <0.001 | 1.32 (1.20, 1.46)  <0.001 | 1.33 (1.20, 1.46)  <0.001 | 1.32 (1.20, 1.46)  <0.001 |
| Q3 | 1.71 (1.57, 1.87)  <0.001 | 1.72 (1.57, 1.87)  <0.001 | 1.91 (1.74, 2.09)  <0.001 | 1.91 (1.74, 2.10)  <0.001 | 1.91 (1.74, 2.10)  <0.001 |
| Q4 | 2.06 (1.89, 2.24)  <0.001 | 2.06 (1.89, 2.24)  <0.001 | 2.31 (2.10, 2.53)  <0.001 | 2.32 (2.11, 2.54)  <0.001 | 2.31 (2.11, 2.54)  <0.001 |
| P for trend | <0.001 | <0.001 | <0.001 | <0.001 | <0.001 |

Model 1: no covariates were adjusted.

Model 2: age, sex, BMI, and smoking status were adjusted.

Model 3: Model 2 plus adjustment for PA, diabetes, CHD, and cancer.

Model 4: Model 3 plus adjustment for ALT, AST, Cr, eGFR, TC, TG, HDL.C, LDL.C, FPG, HbA1c, and UA.

Model 5: Model 4 plus adjustment for use of statins, aspirin, diuretics, beta-blockers, calcium channel blockers, ACEIs/ARBs, and oral hypoglycemic agents.

Abbreviations: PAC, plasma aldosterone concentration; OR, odds ratio; CI, confidence interval.

Other abbreviations, see Table 1.

**Table S11.** Sensitivity analysis of the relationship between PAC with gout was performed after excluding patients with BMI > 30 kg/m^2^

| Exposure | Model 1 | Model 2 | Model 3 | Model 4 | Model 5 |
| --- | --- | --- | --- | --- | --- |
|  | OR (95% CI) P | OR (95% CI) P | OR (95% CI) P | OR (95% CI) P | OR (95% CI) P |
| **Gout** |  |  |  |  |  |
| PAC (per 1-ng/dL increase) | 1.05 (1.04, 1.07)  <0.001 | 1.05 (1.04, 1.07)  <0.001 | 1.5 (1.04, 1.07)  <0.001 | 1.05 (1.04, 1.07)  <0.001 | 1.05 (1.04, 1.07)  <0.001 |
| Quartiles of PAC |  |  |  |  |  |
| Q1 | Reference | Reference | Reference | Reference | Reference |
| Q2 | 1.55 (1.12, 2.16)  <0.001 | 1.54 (1.11, 2.15)  <0.001 | 1.60 (1.15, 2.24)  <0.001 | 1.59 (1.14, 3.24)  <0.001 | 1.60 (1.15, 2.25)  <0.001 |
| Q3 | 2.08 (1.52, 2.86)  <0.001 | 2.06 (1.51, 2.83)  <0.001 | 2.19 (1.60, 3.03)  <0.001 | 2.19 (1.60, 3.03)  <0.001 | 2.21 (1.61, 3.05)  <0.001 |
| Q4 | 2.88 (2.15, 3.92)  <0.001 | 2.83 (2.11, 3.84)  <0.001 | 2.94 (2.18, 4.01)  <0.001 | 2.94 (2.18, 4.02)  <0.001 | 2.95 (2.19, 4.04)  <0.001 |
| P for trend | <0.001 | <0.001 | <0.001 | <0.001 | <0.001 |

Model 1: no covariates were adjusted.

Model 2: age, sex, BMI, and smoking status were adjusted.

Model 3: Model 2 plus adjustment for PA, diabetes, CHD, and cancer.

Model 4: Model 3 plus adjustment for ALT, AST, Cr, eGFR, TC, TG, HDL.C, LDL.C, FPG, HbA1c, and UA.

Model 5: Model 4 plus adjustment for use of statins, aspirin, diuretics, beta-blockers, calcium channel blockers, ACEIs/ARBs, and oral hypoglycemic agents.

Abbreviations: PAC, plasma aldosterone concentration; OR, odds ratio; CI, confidence interval.

Other abbreviations, see Table 1.

**Table S12.** Sensitivity analysis of the relationship between PAC with hyperuricemia was performed after excluding patients with PA

| Exposure | Model 1 | Model 2 | Model 3 | Model 4 | Model 5 |
| --- | --- | --- | --- | --- | --- |
|  | OR (95% CI) P | OR (95% CI) P | OR (95% CI) P | OR (95% CI) P | OR (95% CI) P |
| **Hyperuricemia** |  |  |  |  |  |
| PAC (per 1-ng/dL increase) | 1.04 (1.04, 1.05)  <0.001 | 1.04 (1.04, 1.05)  <0.001 | 1.05 (1.04, 1.06)  <0.001 | 1.05 (1.04, 1.06)  <0.001 | 1.05 (1.04, 1.05)  <0.001 |
| Quartiles of PAC |  |  |  |  |  |
| Q1 | Reference | Reference | Reference | Reference | Reference |
| Q2 | 1.27 (1.17, 1.38)  <0.001 | 1.28 (1.18, 1.39)  <0.001 | 1.36 (1.25, 1.49)  <0.001 | 1.37 (1.25, 1.49)  <0.001 | 1.36 (1.25, 1.49)  <0.001 |
| Q3 | 1.73 (1.60, 1.88)  <0.001 | 1.74 (1.61, 1.89)  <0.001 | 1.96 (1.80, 2.14)  <0.001 | 1.96 (1.80, 2.14)  <0.001 | 1.95 (1.79, 2.13)  <0.001 |
| Q4 | 2.08 (1.92, 2.25)  <0.001 | 2.06 (1.91, 2.24)  <0.001 | 2.32 (2.13, 2.53)  <0.001 | 2.32 (2.13, 2.53)  <0.001 | 2.31 (2.12, 2.52)  <0.001 |
| P for trend | <0.001 | <0.001 | <0.001 | <0.001 | <0.001 |

Model 1: no covariates were adjusted.

Model 2: age, sex, BMI, and smoking status were adjusted.

Model 3: Model 2 plus adjustment for diabetes, CHD, and cancer.

Model 4: Model 3 plus adjustment for ALT, AST, Cr, eGFR, TC, TG, HDL.C, LDL.C, FPG, HbA1c, and UA.

Model 5: Model 4 plus adjustment for use of statins, aspirin, diuretics, beta-blockers, calcium channel blockers, ACEIs/ARBs, and oral hypoglycemic agents.

Abbreviations: PAC, plasma aldosterone concentration; OR, odds ratio; CI, confidence interval.

Other abbreviations, see Table 1.

**Table S13.** Sensitivity analysis of the relationship between PAC with gout was performed after excluding patients with PA

| Exposure | Model 1 | Model 2 | Model 3 | Model 4 | Model 5 |
| --- | --- | --- | --- | --- | --- |
|  | OR (95% CI) P | OR (95% CI) P | OR (95% CI) P | OR (95% CI) P | OR (95% CI) P |
| **Gout** |  |  |  |  |  |
| PAC (per 1-ng/dL increase) | 1.06 (1.04, 1.07)  <0.001 | 1.06 (1.04, 1.07)  <0.001 | 1.06 (1.04, 1.07)  <0.001 | 1.06 (1.04, 1.07)  <0.001 | 1.06 (1.04, 1.07)  <0.001 |
| Quartiles of PAC |  |  |  |  |  |
| Q1 | Reference | Reference | Reference | Reference | Reference |
| Q2 | 1.60 (1.19, 2.15)  <0.001 | 1.59 (1.19, 2.14)  <0.001 | 1.68 (1.25, 2.27)  <0.001 | 1.68 (1.25, 2.27)  <0.001 | 1.69 (1.26, 2.28)  <0.001 |
| Q3 | 2.19 (1.67, 2.91)  <0.001 | 2.17 (1.65, 2.88)  <0.001 | 2.30 (1.74, 3.07)  <0.001 | 2.31 (1.74, 3.07)  <0.001 | 2.31 (1.75, 3.08)  <0.001 |
| Q4 | 3.11 (2.39, 4.08)  <0.001 | 2.99 (2.30, 3.93)  <0.001 | 3.12 (2.39, 4.12)  <0.001 | 3.12 (2.39, 4.12)  <0.001 | 3.12 (2.39, 4.12)  <0.001 |
| P for trend | <0.001 | <0.001 | <0.001 | <0.001 | <0.001 |

Model 1: no covariates were adjusted.

Model 2: age, sex, BMI, and smoking status were adjusted.

Model 3: Model 2 plus adjustment for diabetes, CHD, and cancer.

Model 4: Model 3 plus adjustment for ALT, AST, Cr, eGFR, TC, TG, HDL.C, LDL.C, FPG, HbA1c, and UA.

Model 5: Model 4 plus adjustment for use of statins, aspirin, diuretics, beta-blockers, calcium channel blockers, ACEIs/ARBs, and oral hypoglycemic agents.

Abbreviations: PAC, plasma aldosterone concentration; OR, odds ratio; CI, confidence interval.

Other abbreviations, see Table 1.

**Table S14.** Sensitivity analysis of the relationship between PAC with hyperuricemia was performed after excluding patients with cancer

| Exposure | Model 1 | Model 2 | Model 3 | Model 4 | Model 5 |
| --- | --- | --- | --- | --- | --- |
|  | OR (95% CI) P | OR (95% CI) P | OR (95% CI) P | OR (95% CI) P | OR (95% CI) P |
| **Hyperuricemia** |  |  |  |  |  |
| PAC (per 1-ng/dL increase) | 1.05 (1.04, 1.05)  <0.001 | 1.05 (1.04, 1.05)  <0.001 | 1.05 (1.05, 1.06)  <0.001 | 1.05 (1.05, 1.06)  <0.001 | 1.05 (1.05, 1.06)  <0.001 |
| Quartiles of PAC |  |  |  |  |  |
| Q1 | Reference | Reference | Reference | Reference | Reference |
| Q2 | 1.30 (1.20, 1.40)  <0.001 | 1.29 (1.20, 1.40)  <0.001 | 1.38 (1.27, 1.50)  <0.001 | 1.38 (1.27, 1.50)  <0.001 | 1.38 (1.28, 1.50)  <0.001 |
| Q3 | 1.79 (1.67, 1.93)  <0.001 | 1.80 (1.67, 1.94)  <0.001 | 2.03 (1.88, 2.21)  <0.001 | 2.04 (1.88, 2.21)  <0.001 | 2.07 (1.89, 2.20)  <0.001 |
| Q4 | 2.23 (2.07, 2.40)  <0.001 | 2.22 (2.07, 2.40)  <0.001 | 2.55 (2.35, 2.76)  <0.001 | 2.56 (2.36, 2.77)  <0.001 | 2.55 (2.36, 2.78)  <0.001 |
| P for trend | <0.001 | <0.001 | <0.001 | <0.001 | <0.001 |

Model 1: no covariates were adjusted.

Model 2: age, sex, BMI, and smoking status were adjusted.

Model 3: Model 2 plus adjustment for PA, diabetes, and CHD.

Model 4: Model 3 plus adjustment for ALT, AST, Cr, eGFR, TC, TG, HDL.C, LDL.C, FPG, HbA1c, and UA.

Model 5: Model 4 plus adjustment for use of statins, aspirin, diuretics, beta-blockers, calcium channel blockers, ACEIs/ARBs, and oral hypoglycemic agents.

Abbreviations: PAC, plasma aldosterone concentration; OR, odds ratio; CI, confidence interval.

Other abbreviations, see Table 1.

**Table S15.** Sensitivity analysis of the relationship between PAC with gout was performed after excluding patients with cancer

| Exposure | Model 1 | Model 2 | Model 3 | Model 4 | Model 5 |
| --- | --- | --- | --- | --- | --- |
|  | OR (95% CI) P | OR (95% CI) P | OR (95% CI) P | OR (95% CI) P | OR (95% CI) P |
| **Gout** |  |  |  |  |  |
| PAC (per 1-ng/dL increase) | 1.05 (1.04, 1.06)  <0.001 | 1.05 (1.03, 1.06)  <0.001 | 1.05 (1.03, 1.06)  <0.001 | 1.05 (1.03, 1.06)  <0.001 | 1.04 (1.02, 1.05)  <0.001 |
| Quartiles of PAC |  |  |  |  |  |
| Q1 | Reference | Reference | Reference | Reference | Reference |
| Q2 | 1.46 (1.19, 2.15)  <0.001 | 1.45 (1.11, 1.90)  <0.001 | 1.51 (1.15, 1.98)  <0.001 | 1.51 (1.15, 1.98)  <0.001 | 1.58 (1.20, 2.09)  <0.001 |
| Q3 | 1.91 (1.67, 2.91)  <0.001 | 1.87 (1.46, 2.42)  <0.001 | 1.98 (1.54, 2.57)  <0.001 | 1.98 (1.53, 2.57)  <0.001 | 2.03 (1.57, 2.65)  <0.001 |
| Q4 | 2.66 (2.39, 4.08)  <0.001 | 2.56 (2.02, 3.28)  <0.001 | 2.66 (2.09, 3.42)  <0.001 | 2.66 (2.08, 3.42)  <0.001 | 2.32 (1.81, 3.00)  <0.001 |
| P for trend | <0.001 | <0.001 | <0.001 | <0.001 | <0.001 |

Model 1: no covariates were adjusted.

Model 2: age, sex, BMI, and smoking status were adjusted.

Model 3: Model 2 plus adjustment for PA, diabetes, and CHD.

Model 4: Model 3 plus adjustment for ALT, AST, Cr, eGFR, TC, TG, HDL.C, LDL.C, FPG, HbA1c, and UA.

Model 5: Model 4 plus adjustment for use of statins, aspirin, diuretics, beta-blockers, calcium channel blockers, ACEIs/ARBs, and oral hypoglycemic agents.

Abbreviations: PAC, plasma aldosterone concentration; OR, odds ratio; CI, confidence interval.

Other abbreviations, see Table 1.

**Table S16.** Sensitivity analysis of the relationship between PAC with hyperuricemia was performed after excluding patients with taking diuretics.

| Exposure | Model 1 | Model 2 | Model 3 | Model 4 | Model 5 |
| --- | --- | --- | --- | --- | --- |
|  | OR (95% CI) P | OR (95% CI) P | OR (95% CI) P | OR (95% CI) P | OR (95% CI) P |
| **Gout** |  |  |  |  |  |
| PAC (per 1-ng/dL increase) | 1.04 (1.04, 1.05)  <0.001 | 1.04 (1.04, 1.05)  <0.001 | 1.04 (1.04, 1.05)  <0.001 | 1.05 (1.04, 1.05)  <0.001 | 1.05 (1.04, 1.05)  <0.001 |
| Quartiles of PAC |  |  |  |  |  |
| Q1 | Reference | Reference | Reference | Reference | Reference |
| Q2 | 1.27 (1.17, 1.38)  <0.001 | 1.28 (1.17, 1.39)  <0.001 | 1.28 (1.18, 1.39)  <0.001 | 1.31 (1.20, 1.42)  <0.001 | 1.31 (1.20, 1.42)  <0.001 |
| Q3 | 1.74 (1.61, 1.89)  <0.001 | 1.75 (1.62, 1.90)  <0.001 | 1.76 (1.63, 1.91)  <0.001 | 1.87 (1.72, 2.04)  <0.001 | 1.87 (1.72, 2.04)  <0.001 |
| Q4 | 2.09 (1.93, 2.26)  <0.001 | 2.08 (1.92, 2.25)  <0.001 | 2.09 (1.93, 2.26)  <0.001 | 2.19 (2.02, 2.38)  <0.001 | 2.19 (2.02, 2.38)  <0.001 |
| P for trend | <0.001 | <0.001 | <0.001 | <0.001 | <0.001 |

Model 1: no covariates were adjusted.

Model 2: age, sex, BMI, and smoking status were adjusted.

Model 3: Model 2 plus adjustment for PA, diabetes, and CHD.

Model 4: Model 3 plus adjustment for ALT, AST, Cr, eGFR, TC, TG, HDL.C, LDL.C, FPG, HbA1c, and UA.

Model 5: Model 4 plus adjustment for use of statins, aspirin, beta-blockers, calcium channel blockers, ACEIs/ARBs, and oral hypoglycemic agents.

Abbreviations: PAC, plasma aldosterone concentration; OR, odds ratio; CI, confidence interval.

Other abbreviations, see Table 1.

**Table S17.** Sensitivity analysis of the relationship between PAC with gout was performed after excluding patients with taking diuretics.

| Exposure | Model 1 | Model 2 | Model 3 | Model 4 | Model 5 |
| --- | --- | --- | --- | --- | --- |
|  | OR (95% CI) P | OR (95% CI) P | OR (95% CI) P | OR (95% CI) P | OR (95% CI) P |
| **Gout** |  |  |  |  |  |
| PAC (per 1-ng/dL increase) | 1.06 (1.05, 1.07)  <0.001 | 1.06 (1.04, 1.07)  <0.001 | 1.06 (1.04, 1.07)  <0.001 | 1.06 (1.04, 1.07)  <0.001 | 1.06 (1.04, 1.07)  <0.001 |
| Quartiles of PAC |  |  |  |  |  |
| Q1 | Reference | Reference | Reference | Reference | Reference |
| Q2 | 1.57 (1.18, 2.12)  <0.001 | 1.56 (1.17, 2.11)  <0.001 | 1.56 (1.17, 2.11)  <0.001 | 1.57 (1.17, 2.11)  <0.001 | 1.57 (1.17, 2.12)  <0.001 |
| Q3 | 2.29 (1.74, 3.03)  <0.001 | 2.26 (1.72, 2.99)  <0.001 | 2.27 (1.73, 3.00)  <0.001 | 2.30 (1.75, 3.05)  <0.001 | 2.30 (1.75, 3.06)  <0.001 |
| Q4 | 3.13 (2.42, 4.11)  <0.001 | 3.03 (2.33, 3.97)  <0.001 | 3.03 (2.33, 3.98)  <0.001 | 3.00 (2.30, 3.95)  <0.001 | 3.01 (2.31, 3.96)  <0.001 |
| P for trend | <0.001 | <0.001 | <0.001 | <0.001 | <0.001 |

Model 1: no covariates were adjusted.

Model 2: age, sex, BMI, and smoking status were adjusted.

Model 3: Model 2 plus adjustment for PA, diabetes, and CHD.

Model 4: Model 3 plus adjustment for ALT, AST, Cr, eGFR, TC, TG, HDL.C, LDL.C, FPG, HbA1c, and UA.

Model 5: Model 4 plus adjustment for use of statins, aspirin, beta-blockers, calcium channel blockers, ACEIs/ARBs, and oral hypoglycemic agents.

Abbreviations: PAC, plasma aldosterone concentration; OR, odds ratio; CI, confidence interval.

Other abbreviations, see Table 1.

**Table S18.** E-values for the observed associations between PAC and hyperuricemia.

|  | Model 1 | Model 2 | Model 3 | Model 4 | Model 5 |
| --- | --- | --- | --- | --- | --- |
| PAC (per 1-ng/dL increase) | 1.04  (1.04, 1.05) | 1.04  (1.04, 1.05) | 1.05  (1.04, 1.05) | 1.05  (1.04, 1.05) | 1.04  (1.04, 1.05) |
| E-value for point estimate | **1.24** | **1.24** | **1.28** | **1.28** | **1.24** |

Model 1: no covariates were adjusted.

Model 2: age, sex, BMI, and smoking status were adjusted.

Model 3: Model 2 plus adjustment for PA, diabetes, and CHD.

Model 4: Model 3 plus adjustment for ALT, AST, Cr, eGFR, TC, TG, HDL.C, LDL.C, FPG, HbA1c, and UA.

Model 5: Model 4 plus adjustment for use of statins, aspirin, diuretics, beta-blockers, calcium channel blockers, ACEIs/ARBs, and oral hypoglycemic agents.

Abbreviations: PAC, plasma aldosterone concentration; OR, odds ratio; CI, confidence interval.

Other abbreviations, see Table 1.

**Table S19.** E-values for the observed associations between PAC and gout.

|  | Model 1 | Model 2 | Model 3 | Model 4 | Model 5 |
| --- | --- | --- | --- | --- | --- |
| PAC (per 1-ng/dL increase) | 1.04  (1.04, 1.05) | 1.04  (1.04, 1.05) | 1.05  (1.04, 1.05) | 1.06  (1.04, 1.07) | 1.06  (1.04, 1.07) |
| E-value for point estimate | **1.24** | **1.24** | **1.28** | **1.31** | **1.31** |

Model 1: no covariates were adjusted.

Model 2: age, sex, BMI, and smoking status were adjusted.

Model 3: Model 2 plus adjustment for PA, diabetes, and CHD.

Model 4: Model 3 plus adjustment for ALT, AST, Cr, eGFR, TC, TG, HDL.C, LDL.C, FPG, HbA1c, and UA.

Model 5: Model 4 plus adjustment for use of statins, aspirin, diuretics, beta-blockers, calcium channel blockers, ACEIs/ARBs, and oral hypoglycemic agents.

Abbreviations: PAC, plasma aldosterone concentration; OR, odds ratio; CI, confidence interval.

Other abbreviations, see Table 1.

**3 Supplementary Figures**

**
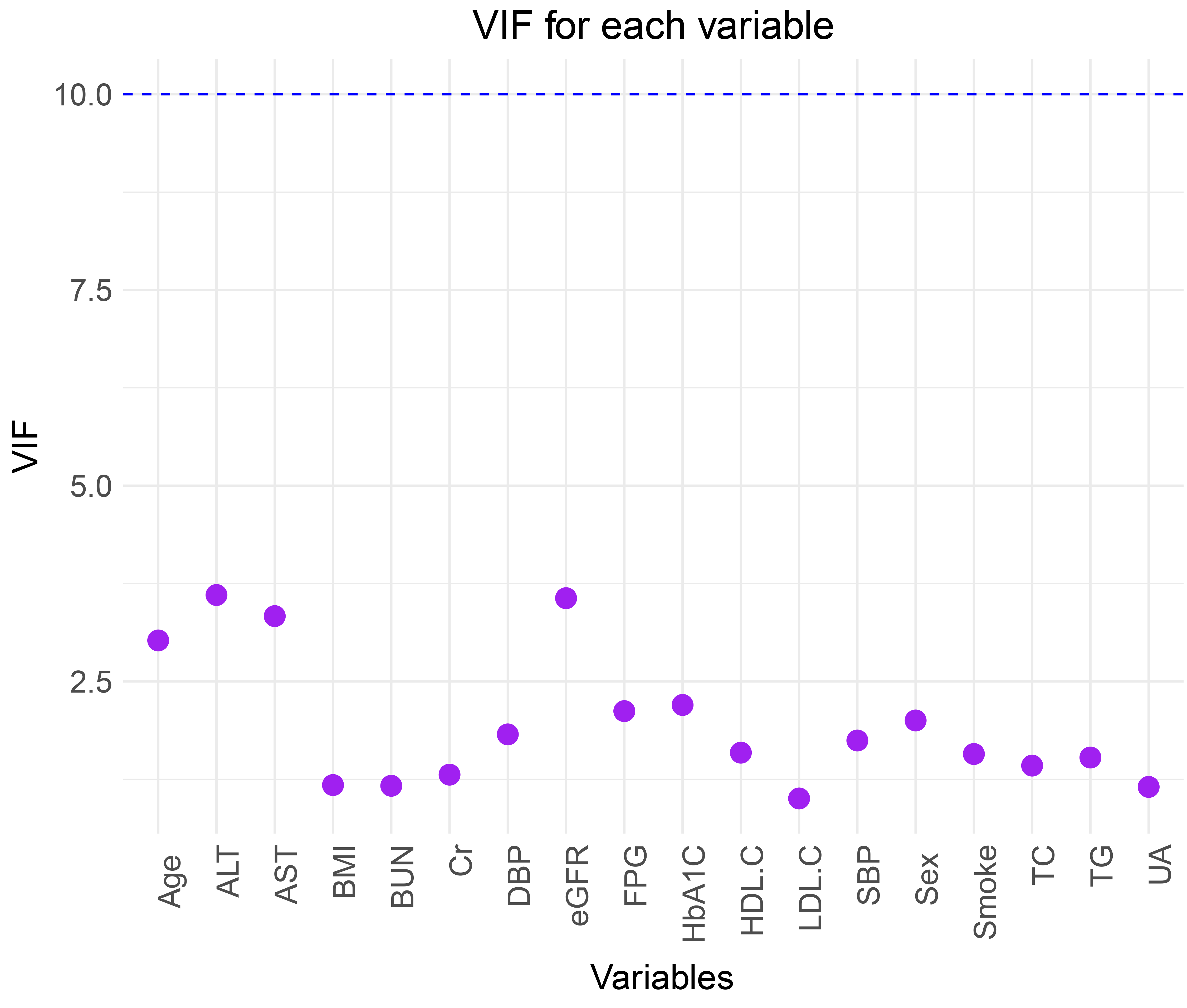
**

**Figure S1** Plots of variance inflation factors for each variable in the research data.


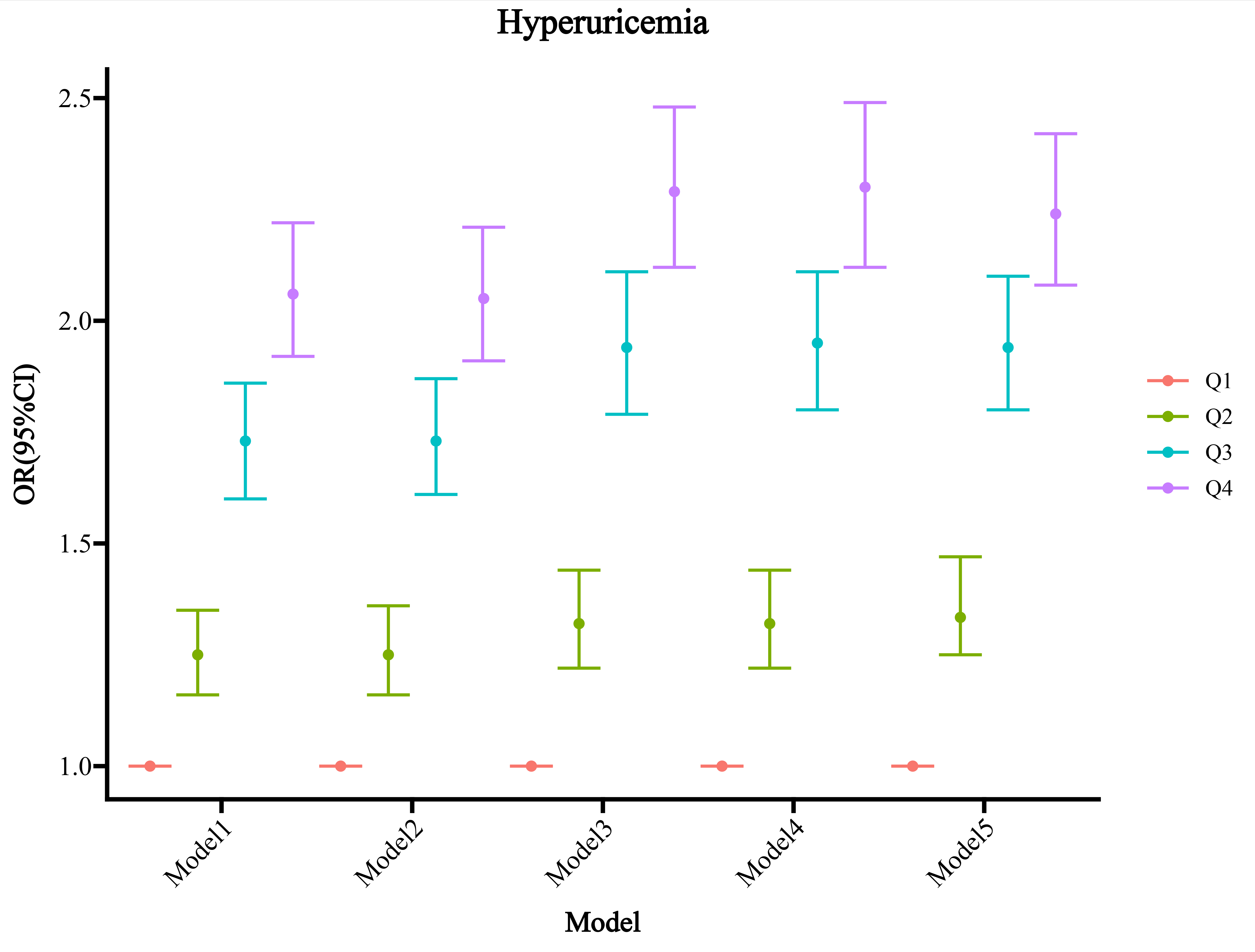


**Figure S2** Multi-model adjusted association between PAC and hyperuricemia


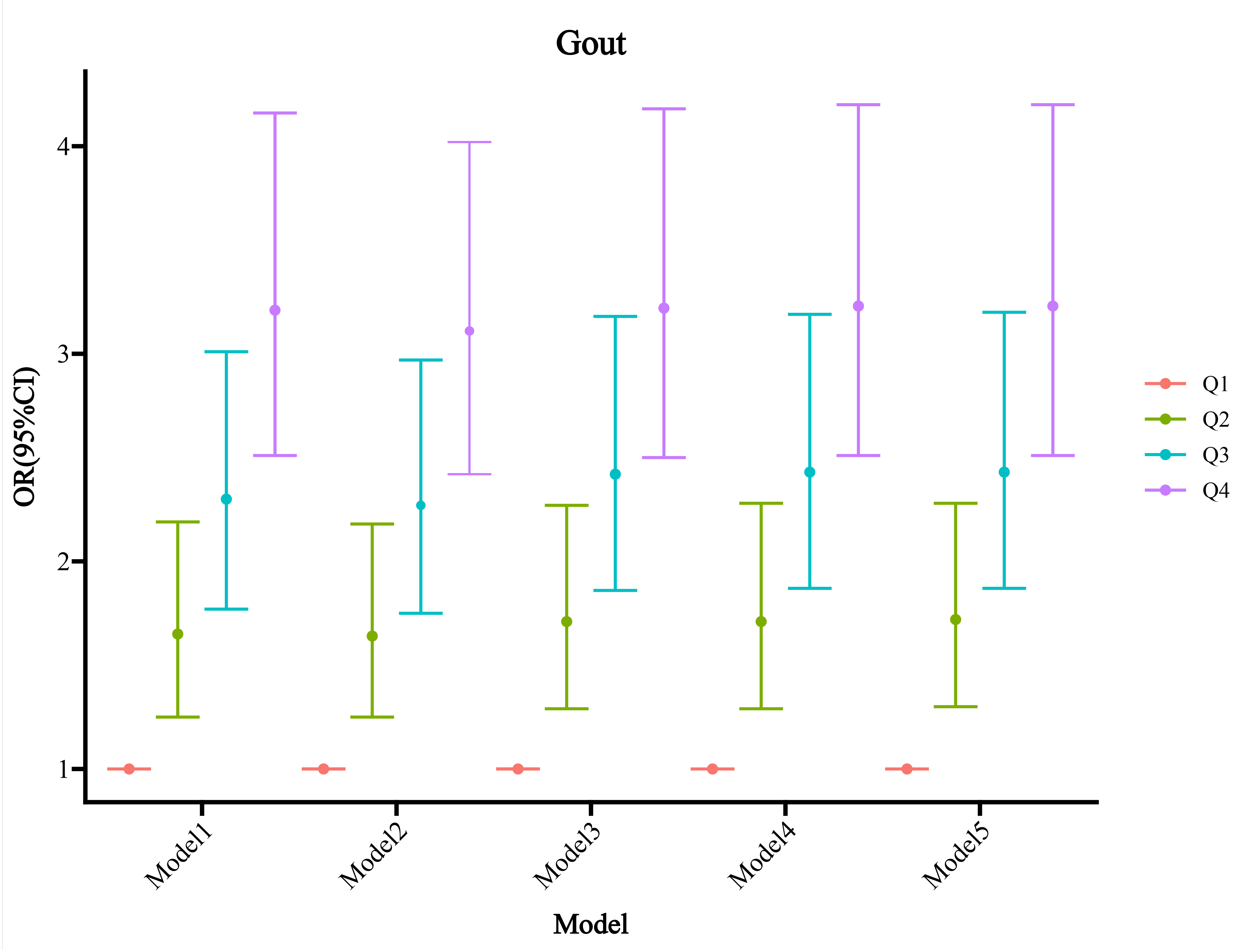


**Figure S3** Multi-model adjusted association between PAC and gout


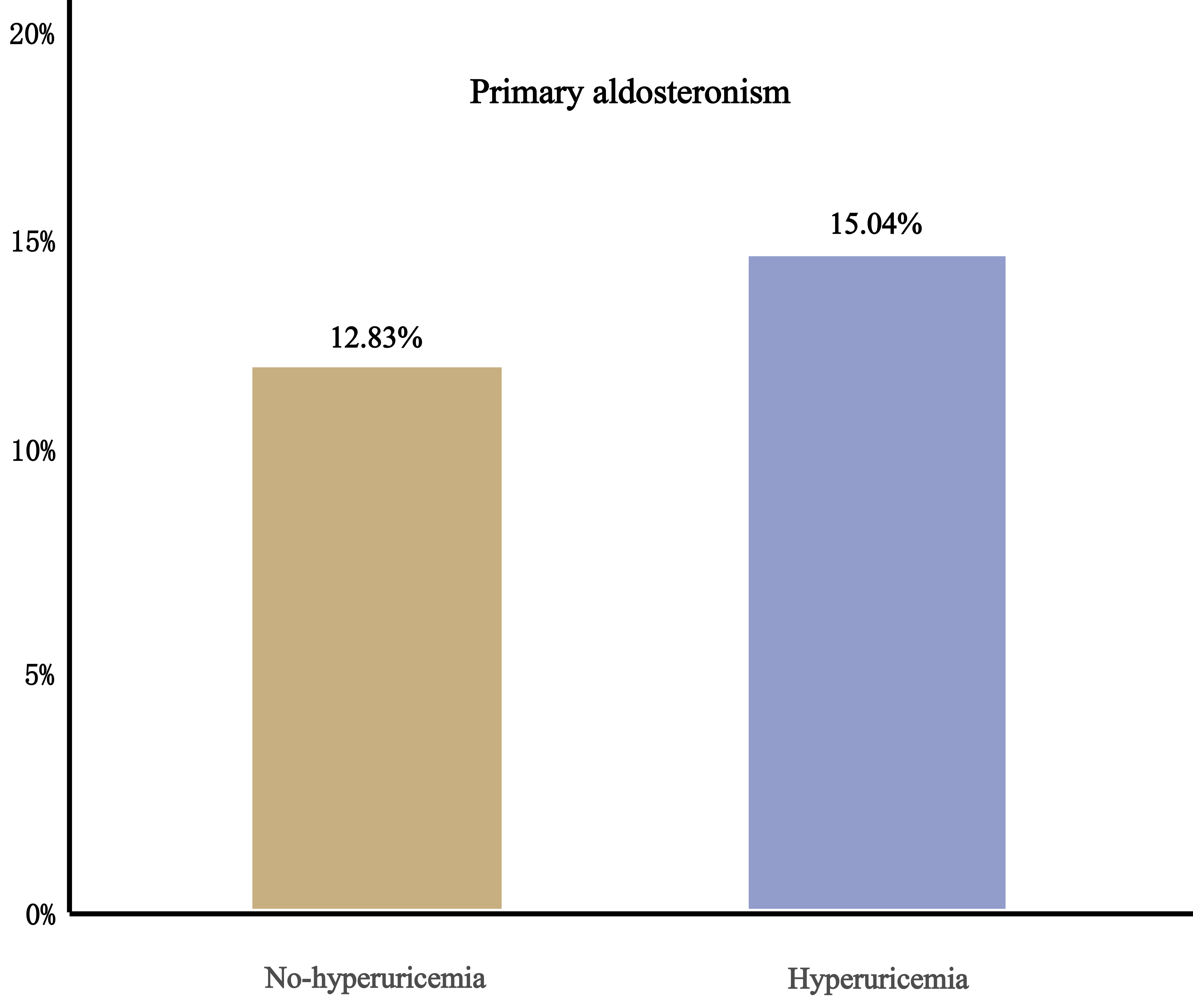


**Figure S4** Prevalence of primary aldosteronism stratified by prevalence of hyperuricaemia


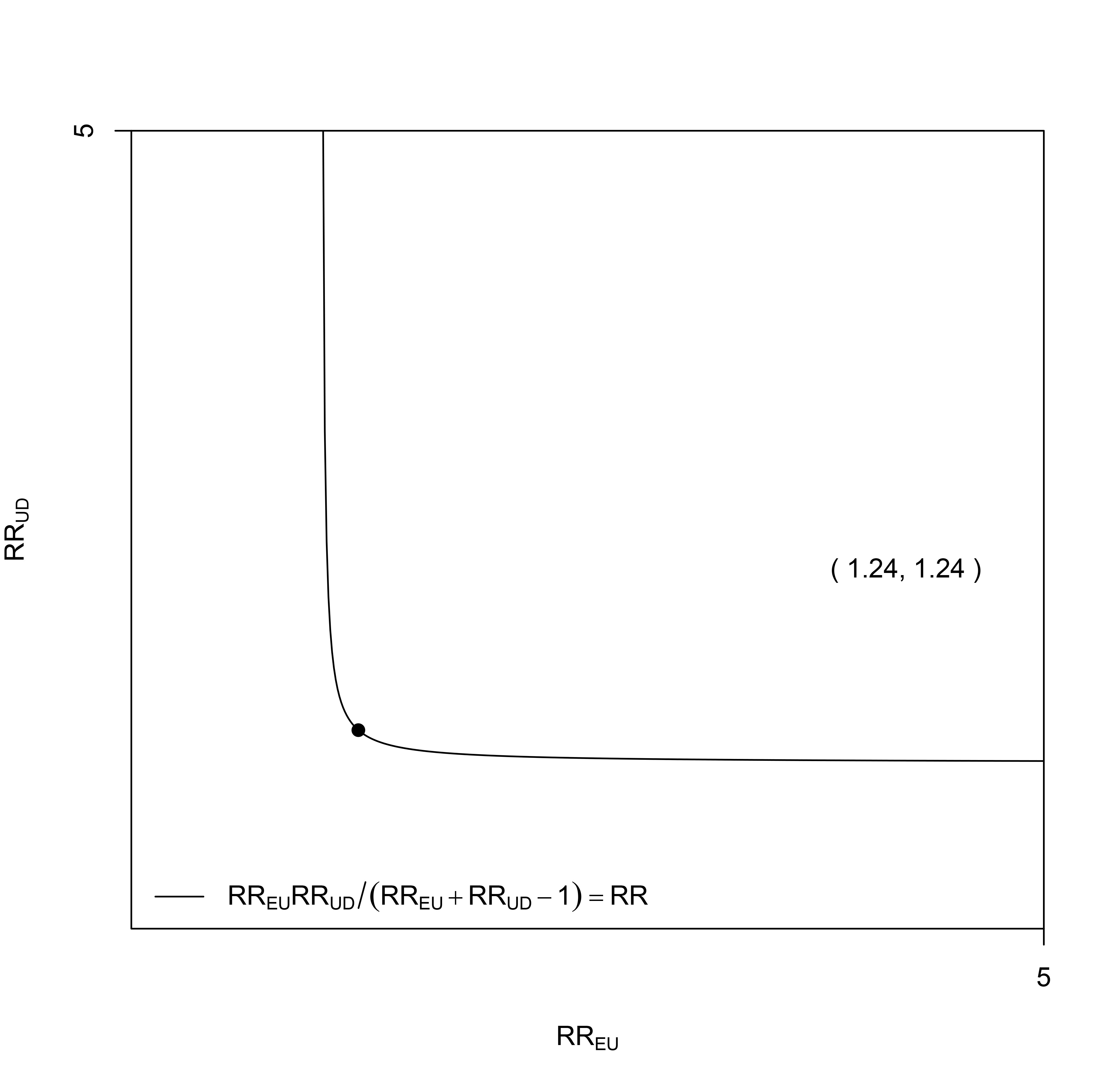


**Figure S5** The E value of the relationship between PAC and hyperuricemia in the fully adjusted model


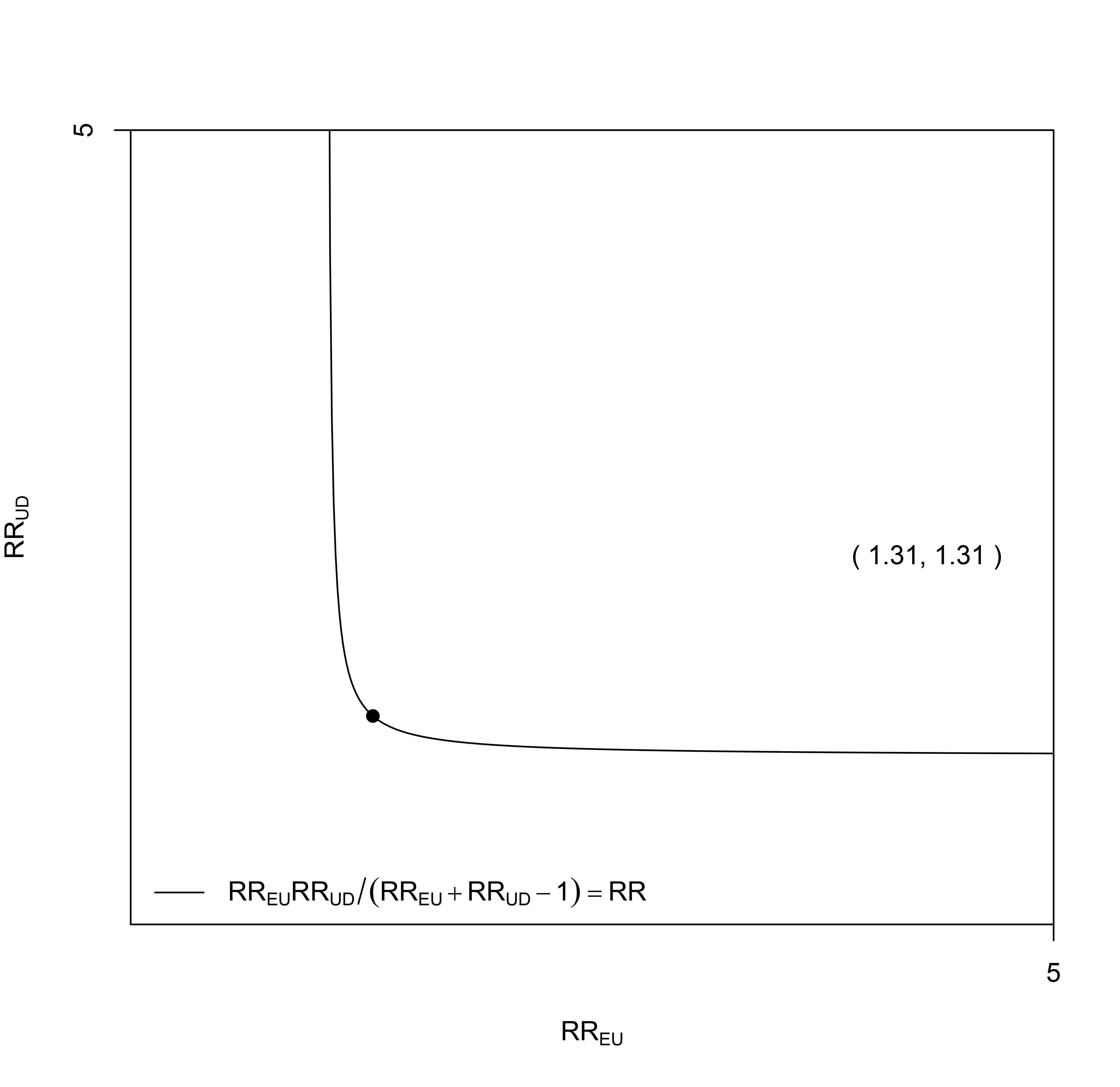


**Figure S6** The E value of the relationship between PAC and gout in the fully adjusted model

**References**

1. Chatterjee S, Khunti K, Davies MJ. Type 2 diabetes. Lancet (London, England). 2017;389(10085):2239-51. Epub 2017/02/14. doi: 10.1016/s0140-6736(17)30058-2. PubMed PMID: 28190580.

2. Song S, Cai X, Hu J, Zhu Q, Shen D, Heizhati M, et al. Correlation between plasma aldosterone concentration and bone mineral density in middle-aged and elderly hypertensive patients: potential impact on osteoporosis and future fracture risk. Frontiers in endocrinology. 2024;15:1373862. Epub 2024/05/29. doi: 10.3389/fendo.2024.1373862. PubMed PMID: 38808106; PubMed Central PMCID: PMCPMC11130431.

3. Cai X, Song S, Hu J, Zhu Q, Shen D, Yang W, et al. Author Correction: Association of the trajectory of plasma aldosterone concentration with the risk of cardiovascular disease in patients with hypertension: a cohort study. Scientific reports. 2024;14(1):9827. Epub 2024/04/30. doi: 10.1038/s41598-024-60563-z. PubMed PMID: 38684882; PubMed Central PMCID: PMCPMC11058239.

4. Farrell GC, Chitturi S, Lau GK, Sollano JD. Guidelines for the assessment and management of non-alcoholic fatty liver disease in the Asia-Pacific region: executive summary. Journal of gastroenterology and hepatology. 2007;22(6):775-7. Epub 2007/06/15. doi: 10.1111/j.1440-1746.2007.05002.x. PubMed PMID: 17565629.
